# Global carbohydrate metabolism is increased and beta hydroxybutyrate decreased in sepsis vulnerable children compared to sepsis tolerant children; a cohort study

**DOI:** 10.21203/rs.3.rs-10732509/v1

**Published:** 2026-09-01

**Authors:** Ruth A Carcillo, Rebecca I Caldino Bohn, Aditya Sriram, Kate F Kernan, Ivan Saraiva, Lina Ghaloul-Gonzalez, Dana Y Fuhrman, Christopher M Horvat, Jeremy R Herrman, Yong Y Han, Trung C Nguyen, Brian Ingram, Robert A Berg, Kathy L Meert, Mark W Hall, Christopher JL Newth, Hernando Gomez, Jerry Vockley, Hyun Jung Park, Joseph A Carcillo

**Affiliations:** 1 University of Pittsburgh School of Medicine, Pittsburgh PA, USA; 2 Graduate School of Public Health, University of Pittsburgh, Pittsburgh PA, USA;; 3 Department of Critical Care Medicine, University of Pittsburgh School of Medicine, Pittsburgh, PA, USA; 4 Department of Pediatrics, University of Pittsburgh School of Medicine, Pittsburgh, PA, USA;; 5 Division of Metabolism and Genetics, Department of Pediatrics, University of Pittsburgh School of Medicine, Pittsburgh, PA, USA;; 6 Division of Critical Care Medicine, Department of Anesthesiology, Children’s Hospital of Philadelphia, Philadelphia, PA, USA;; 7 Division of Critical Care Medicine, Department of Pediatrics, Mercy Children’s Hospital Kansas City, Kansas City, Kansas, MO, USA:; 8 Division of Critical Care Medicine, Department of Pediatrics, Texas Children’s Hospital, Houston, Tx, USA;; 9 Metabolon, Morrisville, NC, USA;; 10 Division of Critical Care Medicine, Department of Pediatrics, Central Michigan University, Detroit, MI, USA;; 11 Division of Critical Care Medicine, Department of Pediatrics, Nationwide Children’s Hospital, Columbus, Ohio, USA;; 12 Division of Critical Care Medicine, Department of Pediatrics, Children’s Hospital of Los Angeles, Los Angeles, CA, USA.

**Keywords:** pediatric sepsis, multiple organ failure, immunometabolism, metabolomics, carbohydrate metabolism, beta hydroxybutyrate, sepsis tolerance, sepsis vulnerability

## Abstract

**Background:**

Sepsis investigators propose tolerance is conferred when the dormancy response increases beta hydroxybutyrate to protect organs; whereas, vulnerability occurs when a ‘metabolic switch’ to aerobic glycolysis decreases beta hydroxybutyrate promoting persistent immune cell activation without organ protection. We previously identified tolerant (PedSep A 2% mortality), susceptible (PedSep B 12% mortality and PedSep C 10% mortality) and vulnerable (PedSep D 34% mortality) phenotypes in the PHENOtyping pediatric sepsis induced Multiple organ failure Study (PHENOMS). We hypothesized that carbohydrate metabolism was increased and beta hydroxybutyrate decreased in PedSep D compared to PedSep A in this study.

**Methods:**

Metabolomics were assayed in biobanked plasma samples previously collected at 24–48 hours of sepsis in 375 children ages 44 weeks post gestation to 17 years, using the Metabolon Discovery Panel. Welch’s t-tests identified differences between PedSep B, C, and D with A.

**Results:**

Compared to PedSep A (n =130) PedSep B had similar carbohydrate metabolism and beta hydroxybutyrate but dysregulated branch chain amino acid metabolism (n = 92; p < 0.05); PedSep C had similar carbohydrate metabolism but decreased beta hydroxybutyrate (n = 100; p< 0.05); and, PedSep D had increased carbohydrate metabolism with decreased beta hydroxybutyrate (n = 53; p < 0.05). Causal inference analysis found direct associations of increased glycolysis with decreased beta hydroxybutyrate, and death.

**Conclusions:**

Only PedSep D had increased carbohydrate metabolism with decreased beta hydroxybutyrate providing rationale for designing clinical trials of low carbohydrate ketogenic nutrition to decrease glycolysis, restore dormancy, and improve outcome in these high-risk children.

## Background

One of five child deaths are attributable to sepsis [1]. During experimental bacterial infection, increased carbohydrate metabolism drives inflammation, while increased fatty acid oxidation and beta hydroxybutyrate levels protect organs from injury [2]. Investigators have suggested that a successful host immunometabolism program against infection requires a balanced defense and defense-induced dormancy response [3]. They propose that sepsis occurs when the host defense and dormancy program response to pathogens is dysregulated; specifically, when carbohydrate metabolism is increased producing immune cell activation with hyperinflammation; and, fatty acid oxidation and beta hydroxy butyrate production is inhibited causing a failed defense dormancy response [2,3].

The Medzhitov laboratory demonstrated that pathogen type elicits specific immunometabolic programs in different sepsis models [4–6]. During Listeria bacterial infection administration of glucose increased mortality, in part related to inhibition of the beta hydroxybutyrate response [4]. Conversely, during Influenza viral infection administration of glucose increased survival [4]. In a cerebral Malaria model, the glycolysis inhibitor 2-deoxyglucose provided protection by preventing microvascular thrombosis [5]. Different sequences of specific pathogen exposures also invoked different immunometabolic programs [6]. Administration of a viral antigen followed by a bacterial antigen produced the secondary Hemophagocytic Lympho Histiocytosis / Macrophage Activation Syndrome hyperinflammatory sepsis phenotype by reprogramming immunometabolism towards CD8 T-cell and interferon gamma independent, aerobic glycolysis dependent Macrophage Activation Syndrome. The glycolysis inhibitor 2-deoxyglucose was lifesaving after this sequence of antigen exposure; however, when viral antigen was followed by viral antigen exposure, or bacterial antigen was followed by bacterial antigen exposure, or bacterial antigen was followed by viral antigen, then 2-deoxyglucose increased mortality [6]. These findings suggested that heterogeneity in immunometabolic reprogramming contributed to heterogeneity in treatment response to carbohydrate supplementation during experimental sepsis. There is a knowledge gap regarding heterogeneity of carbohydrate metabolism and beta hydroxybutyrate in human sepsis [2].

Following the approach of Seymour et al. in the SENECA study [7–10] we previously applied machine learning using 25 clinical bedside variables at 24 hours in children with sepsis and derived four non-synonymous phenotypes named PedSep A, B, C, and D with different inflammation, organ failure, and survival responses [11–13]. Among these four phenotypes, PedSep A patients were relatively tolerant with 2% mortality; PedSep B and PedSep C patients were more susceptible to poor outcome with 12% and 10% mortality rate, respectively; and PedSep D phenotype patients were most vulnerable to poor outcome with 34 % mortality. PedSep D children had the highest inflammation and number of organ failures including renal, hepatic, and hematologic organ failure, with the lowest platelet counts. They also had the highest likelihood to develop the hyper-inflammatory secondary Hemophagocytic Lympho Histiocytosis Macrophage Activation Syndrome [12]. These four agnostic phenotypes provide an opportunity to examine heterogeneity of carbohydrate metabolism and beta hydroxybutyrate responses in pediatric sepsis.

We herein analyzed the global metabolomic response in biobank plasma samples from this same cohort of pediatric sepsis patients [11–12]. We first tested the hypothesis that the vulnerable PedSep D phenotype had increased carbohydrate metabolism and decreased beta hydroxybutyrate compared to the PedSep A phenotype [3]. Second, we tested the hypothesis that PedSep D children had altered metabolites suggestive of mitochondrial dysfunction, decreased fatty acid beta oxidation, and protein catabolism. Third, using causal inference analysis we tested the hypothesis that increased glycolysis was directly causally associated with decreased beta hydroxybutyrate, incomplete beta oxidation of fatty acids and branch chain amino acids, and increased hospital death.

## Methods

### Study design, population, and setting

This study is a secondary analysis of the multicenter PHENOtyping sepsis-induced Multiorgan failure Study (PHENOMS) [11], approved by the University of Utah Institutional Review Board #70976 on 9/22/2014, with ongoing analysis approved by the University of Pittsburgh IRB #20060235, approval date 7/6/2020. All research procedures were in agreement with the ethical principles outlined in the updated Helsinki declaration (available at https://www.wma.net/policies-post/wma-declaration-of-helsinki/, accessed on 4/14/2025). Written informed consent was obtained from one or more parents/guardians for each child. Assent was garnered when the child was able. The prospective cohort included children ages 44 weeks post gestation to 17 years with sepsis in the 7 PICU Eunice Kennedy Shriver National Institutes of Child Health and Human Development Collaborative Pediatric Critical Care Research Network. A previous report using this dataset identified sepsis phenotypes A-D by utilizing k means consensus clustering on 25 bedside variables available at 24 hours [11, 12].

### Metabolomic analysis

Blood was collected and aliquoted in heparinized tubes within 24–48 hours of sepsis; samples were centrifuged for plasma extraction and then frozen and stored at −80°C. Frozen samples were sent to Metabolon (Morrisville, NC) for further analysis. Untargeted metabolomic analyses were performed by ultrahigh performance liquid chromatography-tandem mass spectroscopy in Metabolon’s Discovery Panel. Raw data was extracted, peak values were identified, and data was processed for quality control by Metabolon’s software. Metabolites were identified by comparison with library entries of purified standards, quantified by area under the curve and reported as batch-normalized values. The lowest detection level was imputed as per Metabolon’s standard procedure [14, 15].

### Definitions of organ dysfunction

Organ dysfunction was assessed daily up to 28 days of follow up as previously defined in the IRB approval [11,12]. Briefly, we used the following definitions by organ system: (i) Central nervous system: Glasgow Coma Scale <12 not explained by sedative use; (ii) Cardiovascular: Shock was defined as requirement of vasopressor agent for systolic blood pressure <5^th^ percentile for age; (iii) Pulmonary: PaO_2_/FiO_2_ ratio <300 requiring mechanical ventilation; (iv) Renal: AKI was defined as presence of oliguria (<0.5mL/kg/h) and serum creatinine >1mg/dL or need for renal replacement therapy; (v) Hepatic: alanine aminotransferase >100 U/L and bilirubin >1mg/dL; (vi) Hematologic: platelet count <100,000/mm^3^ and international normalized ratio >1.5.

### Metabolite Differences

Our primary outcome was differences in Metabolon carbohydrate metabolism and beta hydroxybutyrate metabolite response in sepsis phenotypes PedSep B, C, and D compared with PedSep A. Secondary outcomes included differences in Metabolon mitochondrial dysfunction, peroxisomal dysfunction, TCA cycle, branch chain amino acid, acyl carnitine, dicarboxylic acid, urea cycle, glutamate, and organic acid metabolites in sepsis phenotypes PedSep B, C, and D compared to PedSep A. These analyses were performed using Welch’s t-test to assess mean fold-differences with a p value < 0.05 and q-value < 0.05. Findings were considered clinically significant when the average fold- differences were 1.5 - fold or more than PedSep A; or 0.75 – fold or lower than PedSep A.

### Causal Analysis

To identify causal associations between aerobic glycolysis, failed starvation/dormancy response, mitochondrial dysfunction, and hospital death, we performed a Loop Erase Random Walk causal inference analysis [16] (Supplementary Extended Causal Analysis Methods) in the combined PedSep A, B, C, and D cohorts using metabolites that are readily available to practicing clinicians upon ordering bedside acyl carnitine and organic acid profile measurements. These metabolites included lactate and pyruvate (aerobic glycolysis), beta hydroxy butyrate (starvation/dormancy response), acyl carnitines, 3-methyl glutaconate and 3-methylcrotonyl glycine (mitochondrial dysfunction).

## Results

### PedSep A, B, C, and D.

Three hundred and seventy five children enrolled in the PHENOMS study had blood samples analyzed using the Metabolon Global Discovery Panel (PedSep A - n = 130; PedSep B - n = 92; PedSep C - n =100; and PedSep D - n = 53) [11, 12].

### Medzhitov laboratory theory and hypothesis, and primary outcome finding

The theory and the primary hypothesis proposed by the Medzhitov laboratory [3] are shown in Supplementary Figures 1 and 2. The primary outcome findings in Figure 1 demonstrate that compared to the PedSep A, only PedSep D demonstrated increased carbohydrate metabolism with decreased beta hydroxybutyrate. Included in this figure is the previously published chord plots from the PHENOMS study [12] characterizing PedSep A group (2% mortality) as having the least inflammation (CRP levels) and < 2 organ failures with neurologic, cardiovascular or pulmonary involvement; PedSep B group (12% mortality) as having increased inflammation (CRP levels) with multiple organ failure with neurologic, cardiovascular, and pulmonary failure; PedSep C group (10% mortality) as having increased inflammation (CRP and ferritin levels) and < 2 organ failures with cardiovascular or hepatic involvement; and PedSep D (34% mortality) as having increased inflammation (CRP and ferritin levels) and multiple organ failure with neurologic, respiratory, cardiovascular, hepatic, renal, and hematologic dysfunction.

### Metabolites associated with carbohydrate metabolism, and beta hydroxybutyrate

Figure 2 shows a heatmap of the fold difference of carbohydrate metabolism and beta hydroxybutyrate in PedSep B, C, and D compared to PedSep A. Only PedSep D had increased carbohydrate metabolism with increased lactate, pyruvate, pentose pathway, glycogen, disaccharide and oligosaccharide, and aminosugar metabolites as well as decreased beta hydroxybutyrate (p < 0.05). The similar increases in lactate and pyruvate are consistent with aerobic glycolysis in Ped Sep D [17–20]. The decrease in beta hydroxybutyrate is consistent with a failed starvation / dormancy response. PedSep B had similar carbohydrate metabolism and beta hydoxybutyrate, and PedSep C had similar carbohydrate metabolism with decreased beta hydroxybutyrate (p < 0.05). These changes are illustrated in Figures 4–6, and Supplementary Figure 3.

### Metabolites associated with mitochondrial dysfunction, peroxisome dysfunction, and TCA cycle

Figure 3 shows a heatmap of the fold differences in mitochondrial dysfunction, peroxisomal dysfunction, and TCA cycle metabolites in PedSep B, C, and D compared to PedSep A. PedSep B, C, and D all showed increased 3-methyl glutaconate (p < 0.05) suggestive of mitochondrial dysfunction [21**].** PedSep C and D also had increased pristanate and 2- hydroxy phytanate levels (p < 0.05) indicating peroxisomal dysfunction. Measured TCA metabolites including citrate, aconitate, iso-aconitate, iso-citric lactone, alpha ketoglutarate, succinyl carnitine, succinate, fumarate, malate, 2-methyl citrate/homocitrate, and citraconate/glutaconate were increased in PedSep D compared to PedSep A (p < 0.05) [14]. These changes are illustrated Figures 4–6.

### Metabolites associated with decreased fatty acid beta oxidation.

Accumulation of acylcarnitines with dicarboxylic acids is the recognized biochemical signature of a mitochondrial beta oxidation bottle neck as acyl CoA esters that cannot be oxidized are re-esterified to carnitine and exported, while unoxidized long chain fatty acids are shunted to omega oxidation generating dicarboxylic acids [22, 23]. Figure 7 shows a heatmap of fold differences in acyl carnitine levels and Supplemental Figure 4 shows a heatmap of fold differences in dicarboxylic acids in PedSep B, C, and D compared to PedSep A. PedSep B, C, and D show increased acyl carnitines (p <0.05) and dicarboxylic acids (< 0.05) consistent with mitochondrial dysfunction and impaired beta oxidation. These changes are illustrated in Figures 4–6 and Supplementary Figures 5–8.

### Metabolites associated with dysregulated branch chain amino acid metabolism, glutamate metabolism, and urea cycle metabolism

Global amino acid catabolism in skeletal muscle occurs during sepsis hypothetically to provide further energy substrates [24,25]. The branch chain amino acids (BCAA) are of particular interest as their corresponding alpha ketoacids are glucogenic and ketogenic, providing TCA cycle intermediates. Also the combination of alanine and pyruvate with branch chain amino acids generates glutamate and alpha ketoglutarate, further increasing TCA cycle intermediates. Supplementary Figure 9 shows increased BCAA metabolites in PedSep B, C, and D relative to A (p < 0.05). Supplementary Figure 10 shows increased glutamate and urea cycle metabolites in PedSep D relative to A (p < 0.05). Supplementary Figures 11 – 13 illustrate these changes.

### Metabolites in ACGM Technical Standards Acyl Carnitine Profile

To gain insight into whether metabolic profiles observed in PedSep B, C, and D are reminiscent of known metabolic crisis profiles in children with ‘inherited errors of metabolism’, we plotted the PedSep B, C, and D acyl carnitine fold differences on the ACMG (American College of Medical Genetics) Technical Acyl Carnitine Profile template [26] (Figure 7). PedSep B had increased levels of C5.1, 5, 8, 10, and 10.1; as well as 5 and 6 DC (p <0.05). This profile can be similarly seen in Beta Ketothiolase deficiency (BKT), 2-methyl-3 hydroxybutyryl Co A Dehydrogenase deficiency (MHBD), Isovaleric Acidemia (IVA), Short/Branch Chain Acyl CoA Dehydrogenase deficiency (SBCAD), 3 Hydroxy 3 Methyl Glutaryl CoA Lyase deficiency (HMG), and Glutaric Acidemia (GA1) disorders. PedSep C had increased levels of C4, 5, and 5.1 as well as C6 DC (p <0.05). This profile can similarly be seen in Isobutyryl Dehydrogenase Deficiency (IBD), BKT, MHBD, SBCAD, and HMG disorders. PedSep D had increased levels of C3, C4- C10, C12.1, C4–6 DC, and C5 OH (p < 0.05), a profile seen in the above Inborn Errors of Metabolism as well as Propionic Acidemia (PA), Methyl Malonic Acidemia (MMA), SUCLA2 mediated Mitochondrial DNA depletion syndrome which is a defect in succinate CoA ligase (SUCLA), Biotinidase deficiency (BIO), and Medium Chain Acyl CoA Dehydrogenase deficiency (MCAD) disorders.

### Metabolites in ACGM Technical Standards Organic Acid and Amino Acid Profile

We further plotted the PedSep B, C, and D organic and amino acid fold differences using the ACGM Technical Organic Acid and Amino Profile Standards [27,28] (Figure 8). PedSep B had both increased 3- methycrotonyl glycine (p <0.05) and 3- methylglutaconate (p <0.05), a profile seen in 3-methylcrotonyl-coA carboxylase deficiency (3MCC) and 3 methylglutaconyl CoA carboxylase deficiency (3MG), respectively. In addition to these profiles, PedSep C also had increased 3-(4-hydroxyphenyl) lactate (p <0.05) and pristanate and 2-hydroxyphytanate which are seen in tyrosinemia and peroxisomal disorders, respectively. PedSep D had increased organic acid metabolites that confirmed the acyl carnitine profile including MMA, 3MCC, BIO, HMG, MCAD; and, also had increased metabolites seen in urea cycle disorders (UCD), hyperammonemia, hepatic encephalopathy, N-acetyl glutamate synthase deficiency (NAGS), and phenylketonuria (PKU) (p < 0.05).

### Loop Erased Random Walk Causal Inference Analysis of Metabolites and Death

Figure 9 and Supplementary Figure 14 show the abridged and full Directed Acyclic Graphs of the causal inference analysis of the causal associations of lactate, pyruvate, beta hydroxybutyrate, acyl carnitines, and organic acid metabolites with death. Glycolysis defined by increased lactate and pyruvate at 24–48 hours was causally associated with mitochondrial dysfunction (increased 3-methylglutaconate and 3-methylcrotonylglycine), incomplete beta oxidation of fatty acids and branch chain amino acids (elevated tiglyl carnitine), failed starvation response (decreased beta hydroxy butyrate), and subsequent death.

### Immunometabolic Programming in PedSep D

Figure 10 presents a model built by merging previously observed cytokine findings [12] with our new metabolomic findings in PedSep D in the same patient samples. Increased carbohydrate metabolism with increased glycolysis and pentose phosphate pathway activation induces immune activation and decreases beta hydroxybutyrate facilitating unregulated inflammasome activation, resulting in the highest inflammatory cytokine levels and risk for developing secondary Hemophagocytic Lympho Histiocytosis/Macrophage Activation Syndrome, organ toxicity, and death [12].

## Discussion

This analysis establishes that the PedSep D phenotype with the highest inflammation, most organ failures, and highest mortality, had increased carbohydrate metabolism and decreased beta hydroxybutyrate compared to the less inflamed, lower mortality PedSep A phenotype. These data support the translational laboratory data that links this immunometabolic response to worse outcomes [3–6]. Notably, we identified differences in metabolic programming across the sepsis phenotypes that support potential for heterogeneity of carbohydrate treatment effect in pediatric sepsis. The Loop Erased Random Walk causal inference analysis identified increased glycolysis as a direct causal association with the failed starvation response (low beta hydroxybutyrate) and death, supporting dysregulated carbohydrate metabolism as a potential novel therapeutic target in the high-risk PedSep D sepsis phenotype.

Separate reviews [29–35] have proposed that reprogramming of basic metabolic pathways in microbial sepsis may be a novel therapeutic target. During starvation without sepsis, energy balance is maintained when glucagon, catecholamines, and glucocorticoids induce catabolism of glycogen to glucose, protein to amino acids for gluconeogenesis, and lipids to fatty acids to complex with PPAR alpha to induce beta oxidation to provide energy rich metabolites for Acetyl Co A production [36,37]. During sepsis, energy balance is challenged because the host has anorexia leading to starvation, but the gluconeogenesis and beta oxidation responses are inhibited by the inflammatory response [38,39]. Systemic infection, hypoxia, and cytokines increase carbohydrate metabolism inducing aerobic glycolysis in part to provide rapid energy for immune cell activation; and, pentose phosphate pathway activation to provide building blocks for immune cell proliferation. Mitochondrial metabolism is also altered due to reduced pyruvate dehydrogenase complex activity from HIF 1a increased expression of pyruvate dehydrogenase kinase decreasing pyruvate shuttling into the mitochondria for energy production [40–46]. Mitochondrial DNA and proteins of the electron transport chain display physical damage likely from reactive oxygen species, leading to decreased ATP/ADP ratios [47–50]. Decreased expression of carnitine palmitoyl transferase 1 (CPT1), a key enzyme that allows long chain fatty acids to enter mitochondria [51,52], and decreased PPAR alpha levels lead to inhibition of beta oxidation of fatty acids and decreased beta hydroxybutyrate (a class I histone deacetylase inhibitor) which otherwise provide energy, protect against reactive oxygen species induced organ damage, and inhibit inflammasome activation [53–63]. Because skeletal muscle and the brain use beta hydroxy butyrate as an important energy source, the lack of increased beta hydroxy butyrate is proposed to trigger the protein catabolism response [64–66]. As a result of relative inability to use energy rich metabolites of amino acid and lipid catabolism, their toxic unused metabolites accumulate and induce further organ injury including hyperammonemia and hepatic encephalopathy. Investigators have targeted metabolic reprogramming early in the failed starvation response of sepsis using histone deacetylase inhibitors to attenuate pathologic glycolysis [68–71]; and, metformin to inhibit HIF1a and activate AMPK to reprogram back to fatty acid oxidation [72–75]. A Phase I/II Metformin trial for adult septic shock has been recently completed and is in data analysis (NCT05900284) [76].

We evaluated this overall sepsis metabolism construct, that had been previously developed without attention to heterogeneity carbohydrate metabolism response, in our four non-synonymous pediatric sepsis phenotypes. We found that PedSep B, C, and D phenotypes had increased 3-methylgluconate compared to PedSep A suggesting that mitochondrial dysfunction contributed to poor overall outcome. Although the PedSep B children showed no difference in carbohydrate metabolism or beta hydroxy butyrate; they did have increased acyl carnitine levels that have been seen in disorders of branch chain amino acid metabolism which can lead to organ dysfunction during metabolic crises. The mainstay of treatment for metabolic crises in children with these disorders is continuous glucose infusion to induce anabolism, restriction of leucine and isoleucine, and supplementation with carnitine and mitochondrial vitamins including thiamine and riboflavin to reduce further production of toxic metabolites [77]. The PedSep C children showed similar carbohydrate metabolism, lactate, and pyruvate; however, beta hydroxybutyrate was decreased. Pristanate and 2 -hydroxyphytanate were increased, consistent with reduced alpha oxidation and beta oxidation of fatty acids seen with peroxisomal disorders. These metabolic crises are treated by holding all protein-containing feeds for 24–48 hours, providing continuous glucose infusion to induce anabolism, MCT oil to bypass the alpha and beta oxidation defects, and hydroxyphytanate restricted nutrition to reduce toxic metabolites (e.g., isobutyryl-CoA, methylcrotonyl-CoA) [77].

Only the PedSep D group had increased carbohydrate metabolism with equivalently increased lactate and pyruvate consistent with a metabolic switch to aerobic glycolysis leading to decreased beta hydroxybutyrate with increased protein catabolism. Disorders of pyruvate metabolism also show increased lactate and pyruvate and are managed during metabolic crisis with less rather than more glucose and thiamine to reduce lactate production. Low carbohydrate ketogenic diets are particularly helpful in children with Pyruvate Dehydrogenase Deficiency [77]. In severe cases accumulating metabolites and CoA-ester or substrate imbalances can inhibit N-acetylglutamate synthase or carbamyl phosphate synthetase, producing a secondary hyperammonemia. The PedSep D children had increased metabolites seen in hyperammonemia and urea cycle disorders treatable with ammonia scavenging therapies [77].

Regarding this immunometabolic nutrition approach to PedSep D, a recent Phase II adult septic shock trial [78] reported that 14 days of a eucaloric low carbohydrate ketogenic diet compared to a standard high carbohydrate critical care diet; 1) reduced lactate levels and need for insulin, suggesting that low carbohydrate / higher fat nutrition can inhibit carbohydrate metabolism and aerobic glycolysis [79]; 2) increased beta hydroxy butyrate, showing low carbohydrate nutrition can increase fatty acid oxidation [80–82]; 3) increased organ support free days, demonstrating the ability of increased beta hydroxybutyrate to induce sepsis tolerance [2–4]; and, 4) quelled T-cell activation and pro-inflammatory macrophage cytokine production, supporting a role for nutritional modulation of the hyperinflammatory response [63]. A Phase I trial in adult septic shock reported exogenous intravenous beta hydroxybutyrate infusion reduced lipid catabolism and glucose production, supporting the hypothesis that beta hydroxybutyrate could also protect organ function by reducing production of toxic lipid metabolites and reducing aerobic glycolysis [83, 84].

The main strengths of our study are that we used a large multicenter cohort conferring generalizability in US Pediatric Intensive Care Units; and machine learning derived agnostic, non-synonymous phenotypes limiting bias in identification of tolerant, susceptible, and vulnerable phenotypes to assess heterogeneity in carbohydrate metabolism. In support of our findings, two studies have reported low beta hydroxybutyrate levels in newborns and adults with poor sepsis outcomes, [2, 84] and several pediatric and adult sepsis metabolomic studies have reported reduced beta oxidation, increased acyl carnitines, and amino acid catabolism associated with adverse outcomes [85–96].

There are limitations in our study. There were no critically ill or non-critically ill control groups limiting comparisons to sepsis tolerance. Metabolomic responses change over time and our findings are limited to the 24 – 48 hours after sepsis diagnosis [97]. The standard of clinical care for management of metabolic crises in children with ‘inborn errors of metabolism’ is to do so based on 24-hour metabolic profiles and then to repeat these profiles every 24 – 72 hours to document resolution. Because we used a Global Discovery Panel we do not have metabolites of complete pathways, nor do we have insight into single cell or organ level metabolomics. We further acknowledge potential confounding by severity as metabolite differences may be attributable to variability in severity of illness and clinical care characteristics among the phenotypes [12].

## Conclusion

We observed heterogeneity of carbohydrate metabolism response in pediatric sepsis with a metabolic program of increased carbohydrate metabolism and decreased beta hydroxybutyrate in children identified in the vulnerable PedSep D phenotype within 48 hours of sepsis onset. We also identified direct causal association between increased glycolysis and decreased beta hydroxybutyrate, and death. This provides background, impetus, rationale, feasibility, and an enrollment enrichment strategy for designing clinical trials to test the ability of metabolic therapies such as metformin or low carbohydrate ketogenic nutrition to reduce aerobic glycolysis, restore dormancy, and improve outcome in these high-risk pediatric sepsis patients.

## Supplementary Material

1

Supplementary Files

This is a list of supplementary files associated with this preprint. Click to download.

- IncreasedCarbohydrateMetabolismCriticalCareSupplemntaryMaterial.docx

## Figures and Tables

**Figure 1 -:**
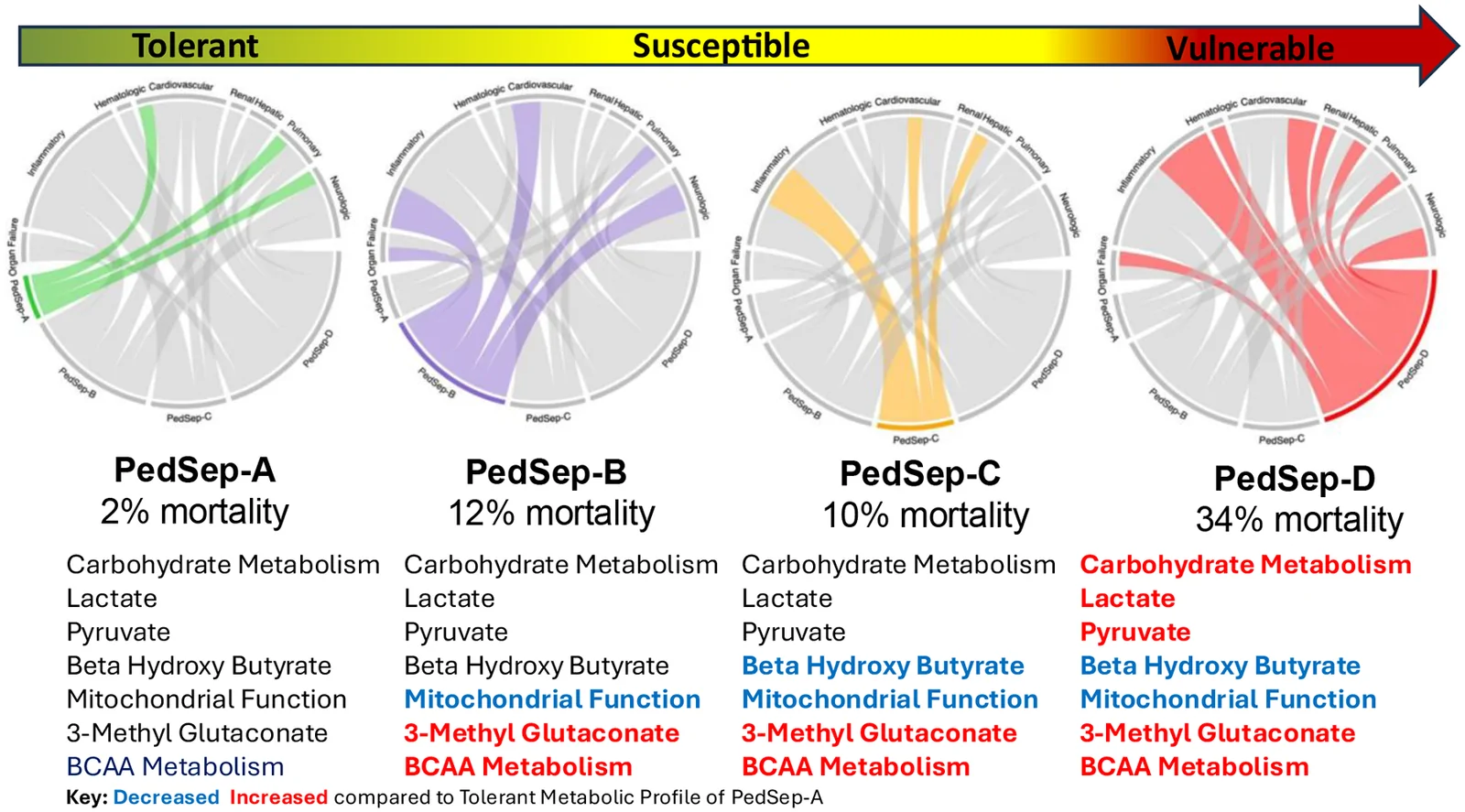
Representation of metabolic findings in tolerant group PedSep A, susceptible groups PedSep B and C, and vulnerable group PedSep D children. The chord plots represent the distribution of proportion above the mean (12). Chords connect from a phenotype to a category if the group mean involvement differs from the overall mean for the entire cohort. being specifically higher for: inflammation = low temperature, high temperature, max CRP, max Ferritin; Organ Failure = total OFI; Pulmonary = pulmonary OFI; Cardiovascular = high heart rate, low systolic blood pressure, cardiovascular OFI; Renal = High creatinine, renal OFI; Hepatic = hepatic OFI; Hematologic = Low hemoglobin, platelets, hematologic OFI, Neurologic = Low Glasgow Coma Score, central nervous system OFI.

**Figure 2 -:**
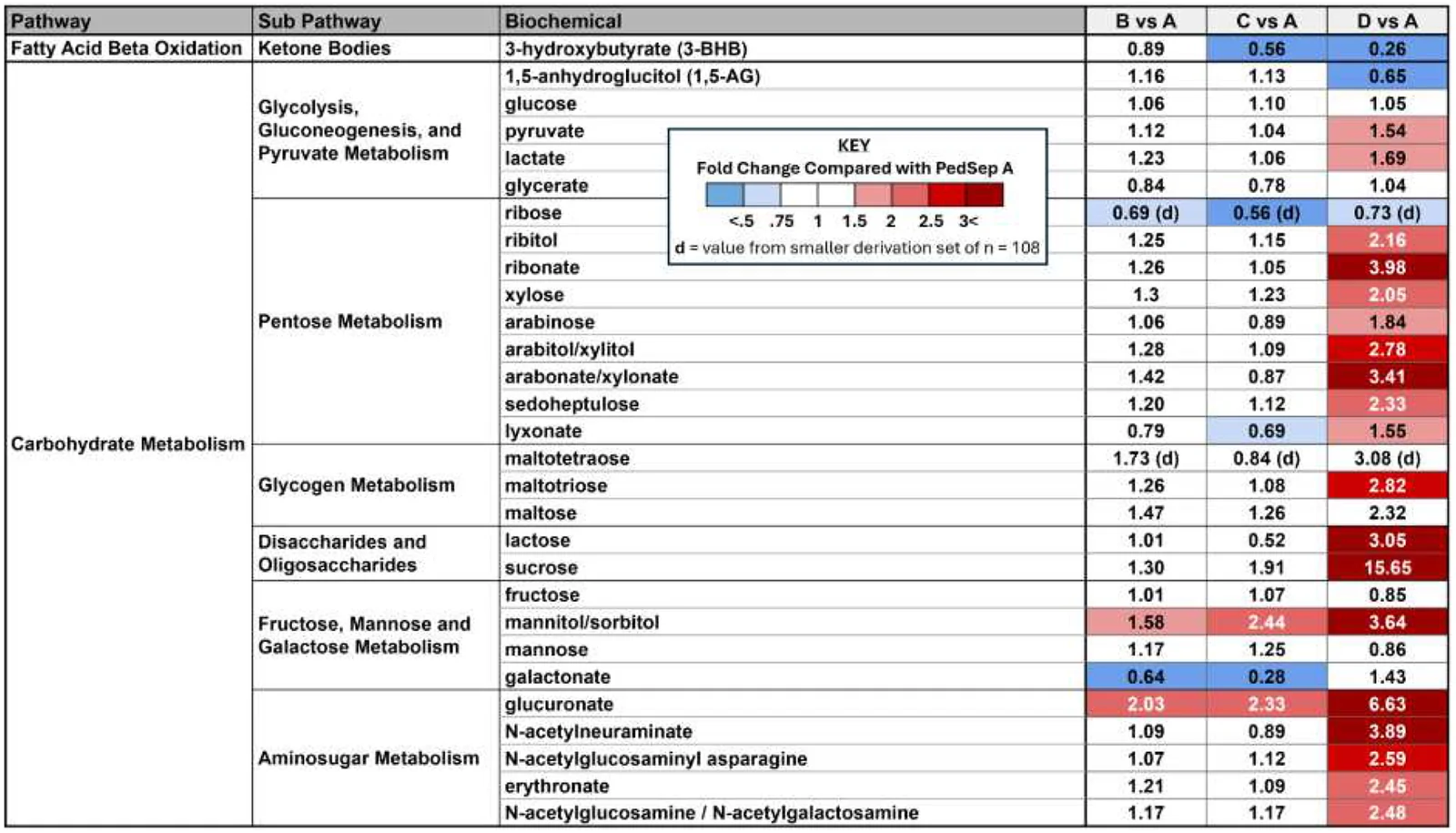
Carbohydrate and beta hydroxy butyrate pathway, sub-pathway, and metabolite fold differences between PedSep B vs A, C vs A, and D vs A. Red represents greater than or equal to 1.5-fold increase and blue is less than or equal to 0.75-fold of A with a P < 0.05 based on Welch’s t-test. d – measured in limited set only (Ped Sep A n = 27; PedSep B n= 27; PedSep C n = 27; PedSep D n = 27).

**Figure 3 -:**
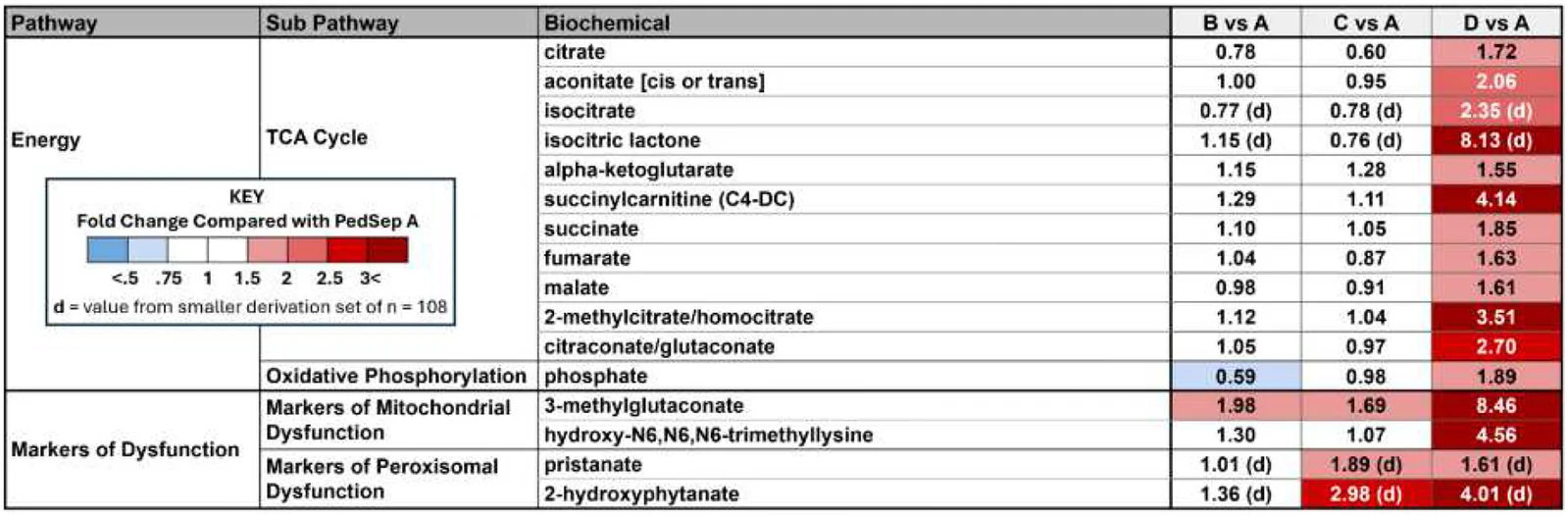
Tricarboxylic acid cycle intermediates and mitochondrial and peroxisomal dysfunction markers pathway, sub-pathway, metabolite fold differences between PedSep B vs A, C vs A, and D vs A. Red represents greater than or equal to 1.5-fold increase and blue is less than or equal to 0.75-fold of A with P < 0.05 based on Welch’s t-test. d.- measured in limited set only (PedSepA n = 27; PedSepB n = 27; PedSep C n = 27; PedSep D n =27).

**Figure 4 -:**
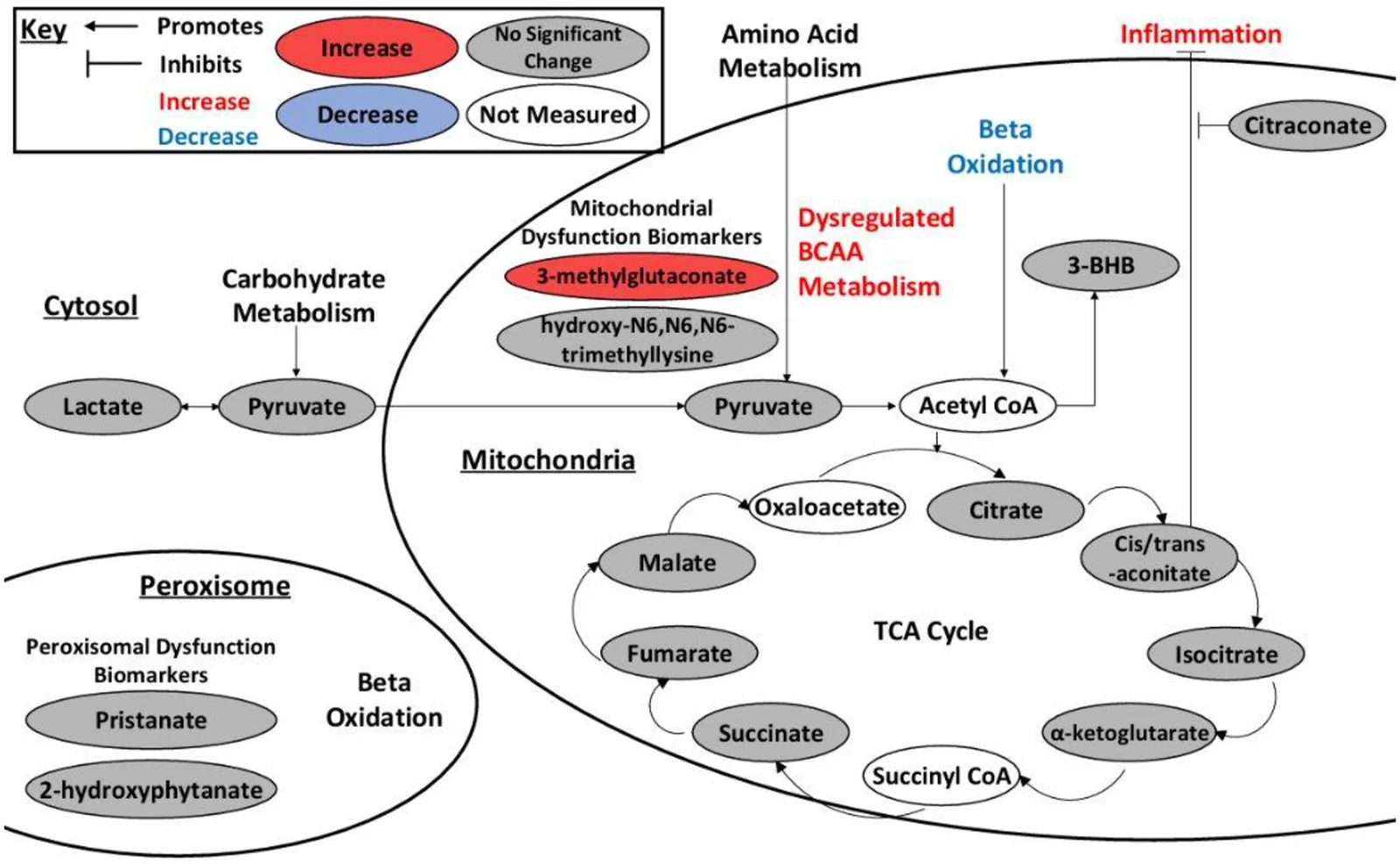
Illustration of dysregulated metabolism in susceptible group B compared to tolerant group A. Red is increased and blue is decreased,

**Figure 5 -:**
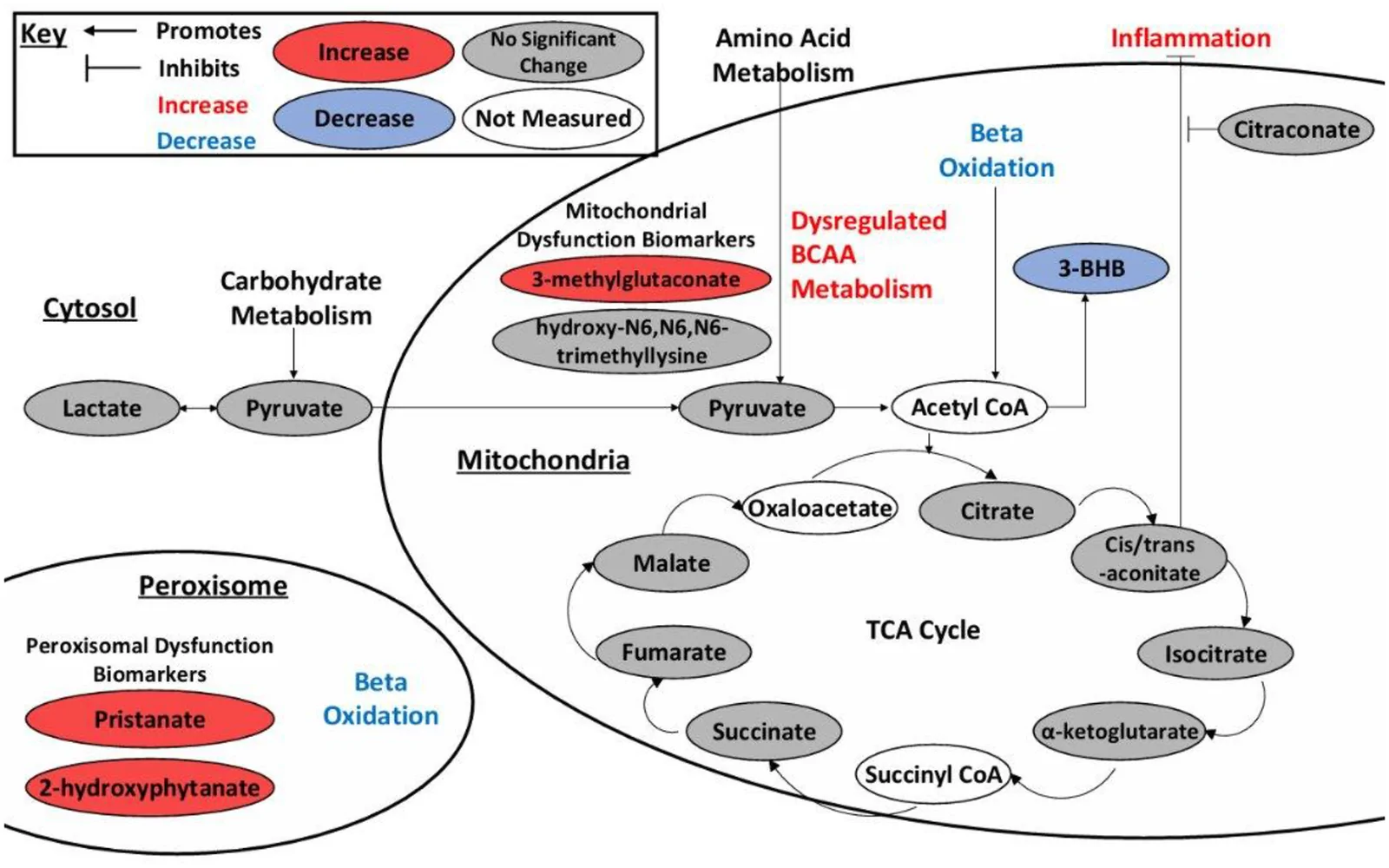
Illustration of dysregulated metabolism in susceptible group C compared to tolerant group A. Red is increased and blue is decreased.

**Figure 6 -:**
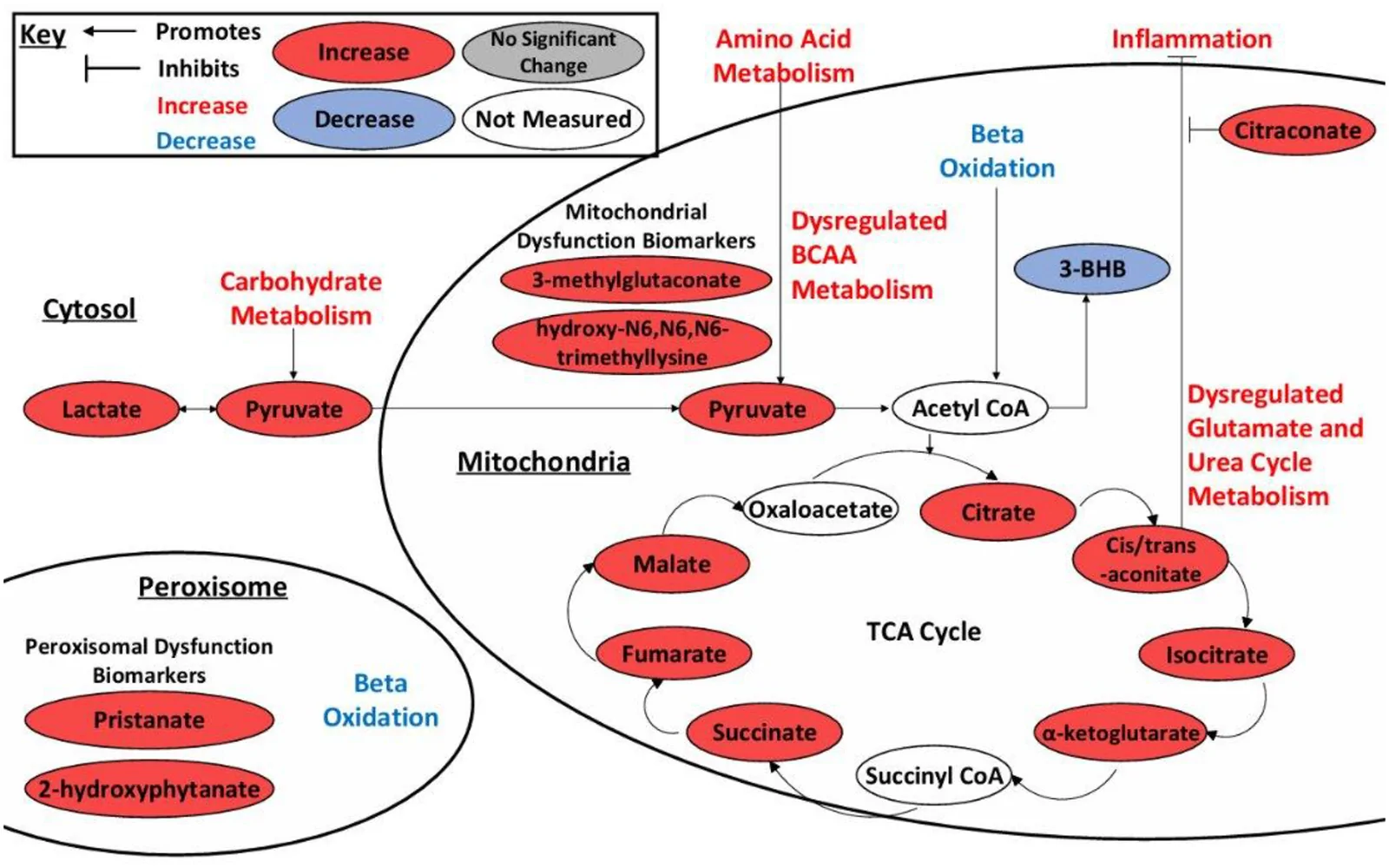
Illustration of dysregulated metabolism in vulnerable group D compared to tolerant group A. Red is increased and blue is decreased.

**Figure 7 -:**
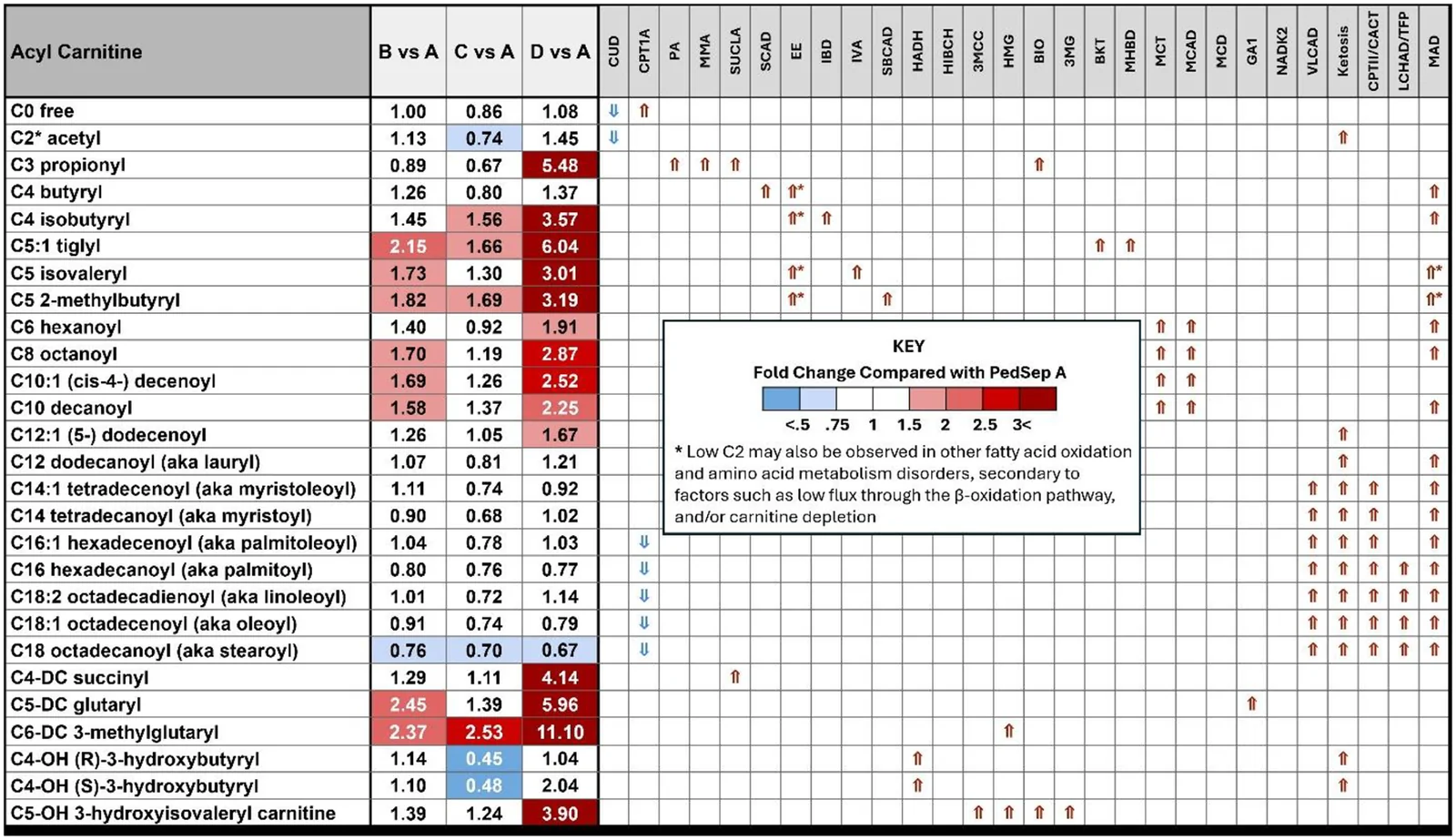
Acyl carnitine profile [26] fold differences between B and A, C and A, and D and A groups. Red represents greater than or equal to 1.5 - fold increase and blue is less than or equal to 0.75 of A with a P < 0.05 based on Welch’s t-test. Red arrows show increased carnitine metabolites and blue arrows show decreased metabolites also seen in specific inborn errors of immunity. CUD **–** Carnitine Uptake Defect; CPT 1A - Carnitine Palmitoyltransferase 1A Deficiency; PA – Propionic Acidemia; MMA – Methyl Malonic Acidemia; SUCLA - SUCLA2-related Mitochondrial DNA Depletion Syndrome; SCAD - Short-Chain Acyl-CoA Dehydrogenase Deficiency; EE - Ethylmalonic Encephalopathy, IBD - Isobutyryl-CoA Dehydrogenase Deficiency, IVA - Isovaleric Acidemia; SBCAD - Short/Branched-Chain Acyl-CoA Dehydrogenase Deficiency; HADH - 3-Hydroxyacyl-CoA Dehydrogenase Deficiency; HIBCH -3-Hydroxyisobutyryl-CoA Hydrolase Deficiency; 3MCC - 3-Methylcrotonyl-CoA carboxylase deficiency; HMG -3-Hydroxy-3-methylglutaryl-CoA lyase deficiency; BIO - Biotinidase deficiency; 3MG - 3-Methylglutaconic Aciduria; BKT - β-Ketothiolase Deficiency; MHBD - 2-Methyl-3-Hydroxybutyryl-CoA Dehydrogenase Deficiency; MCT - Medium chain triglycerides; MCAD - Medium-chain acyl-CoA dehydrogenase deficiency; MCD - Malonyl-CoA Decarboxylase Deficiency; GA1 - Glutaric Acidemia Type I; NADK2 - NAD Kinase 2 Deficiency; VLCAD - Very-Long-Chain Acyl-CoA Dehydrogenase Deficiency; CPTII/CACT - Carnitine Palmitoyltransferase II Deficiency / Carnitine–Acylcarnitine Translocase Deficiency; LCHAD/TFP - Long-chain 3-hydroxyacyl-CoA dehydrogenase deficiency/Trifunctional protein deficiency;; MAD - Multiple acyl-CoA dehydrogenase deficiency.

**Figure 8 -:**
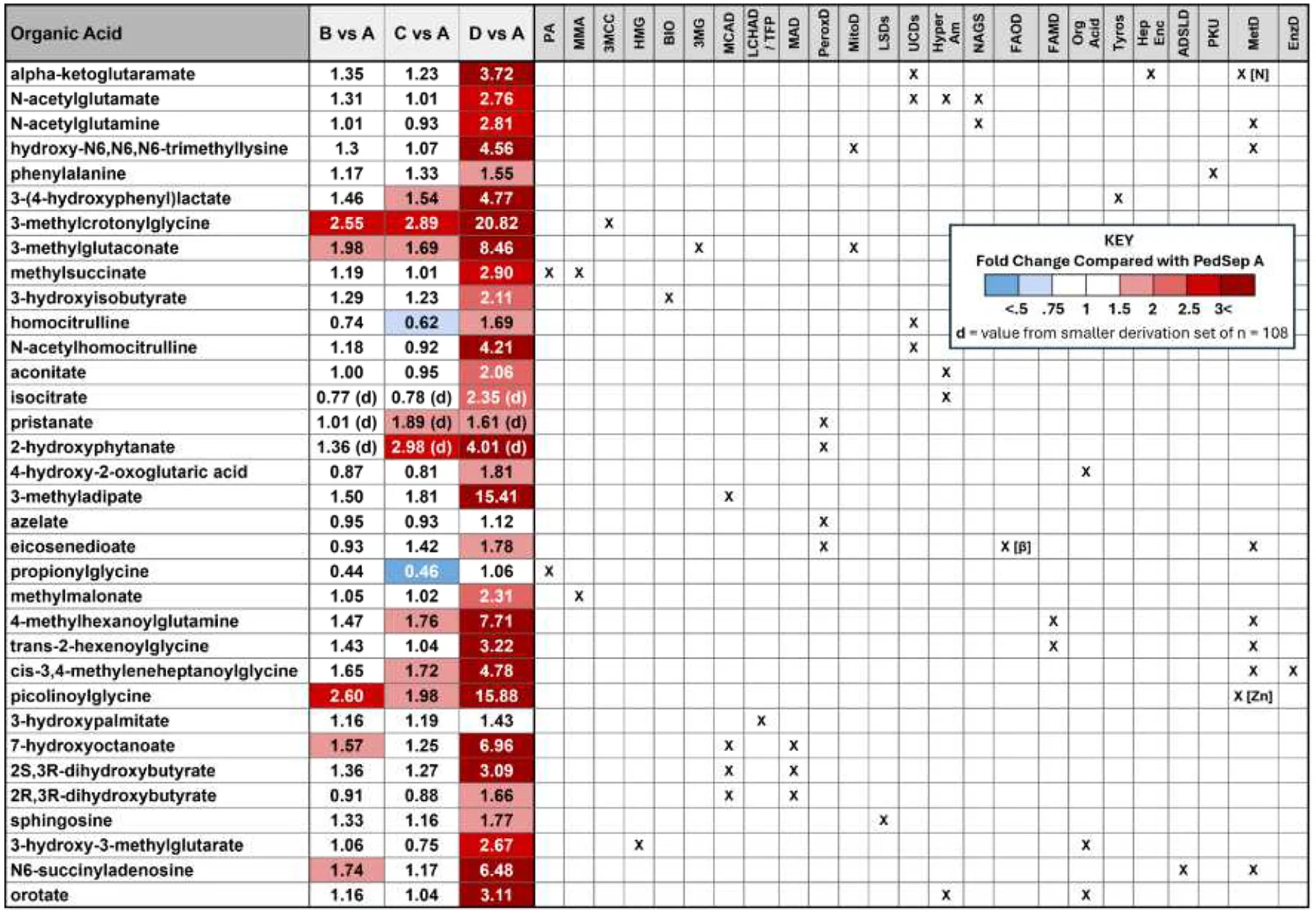
Organic Acid [27] and [28] Amino Acid Profile fold differences between B and A, C and A, and D and A groups. Red represents greater than or equal to 1.5 - fold increase and blue is less than or equal to 0.75 of A with a P < 0.05 and a q < 0.05 based on Welch’s t-test. The x indictes metabolite seen in specific inborn errors of immunity. PA - Propionic Acidemia; MMA – Methyl Malonic Acidemia; 3MCC - 3-Methylcrotonyl-CoA carboxylase deficiency; HMG 3-Hydroxy-3-methylglutaryl-CoA lyase deficiency; BIO - Biotinidase deficiency; 3MG; MCAD - Medium-chain acyl-CoA dehydrogenase deficiency; LCHAD/TFP - Long-chain 3-hydroxyacyl-CoA dehydrogenase deficiency / Trifunctional protein deficiency; MAD - Multiple acyl-CoA dehydrogenase deficiency; PeroxD – Peroxisomal Disorder; MitoD – Mitochondrial Disorder; LSDs - Lysosomal Storage Disorders; UCDs – Urea Cycle Disorders; Hyperam - Hyperammonemia; NAGS - N-acetylglutamate synthase deficiency; FAOD - Fatty-acid oxidation disorders; FAMD - fatty-acid metabolism disorders; Org Acid **–** Organic Acidemia; Tyros - Tyrosinemia; Hep Enc **–** Hepatic Encephalopathy; ADSLD - Argininosuccinate lyase deficiency; PKU - Phenylketonuria; MetD **–** Metabolic Disorder; EnzD **–** Enzyme Deficiency.

**Figure 9 -:**
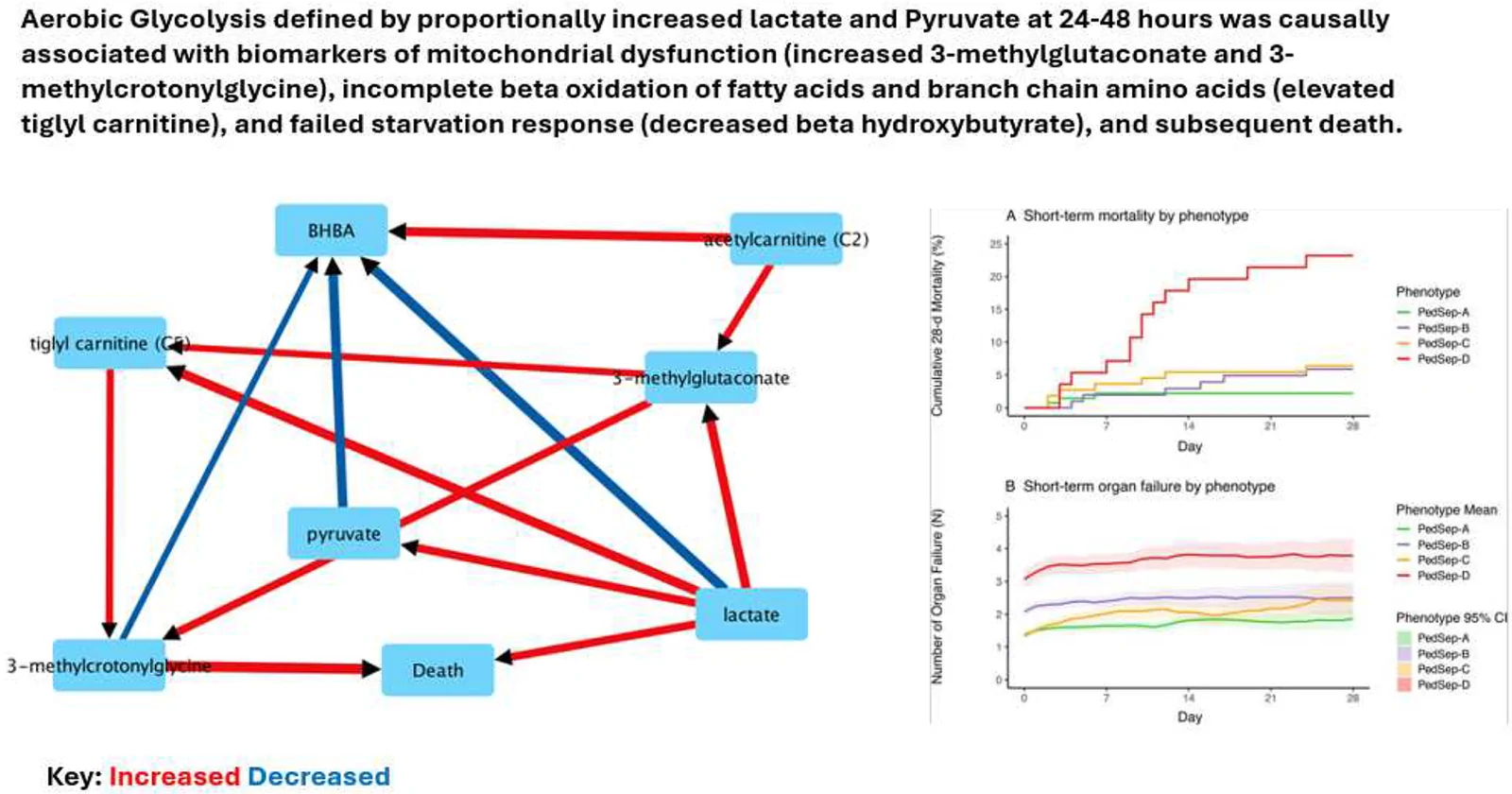
Abridged causal inference analysis of direct associations between metabolites and hospital death in the combined cohort of PedSep A, B, C, and D. Arrows show directionality of causal associations. Red is increased and blue is decreased. The time course of death and organ failure is represented according to group membership with 95% confidence interval shaded (12).

**Figure 10 -:**
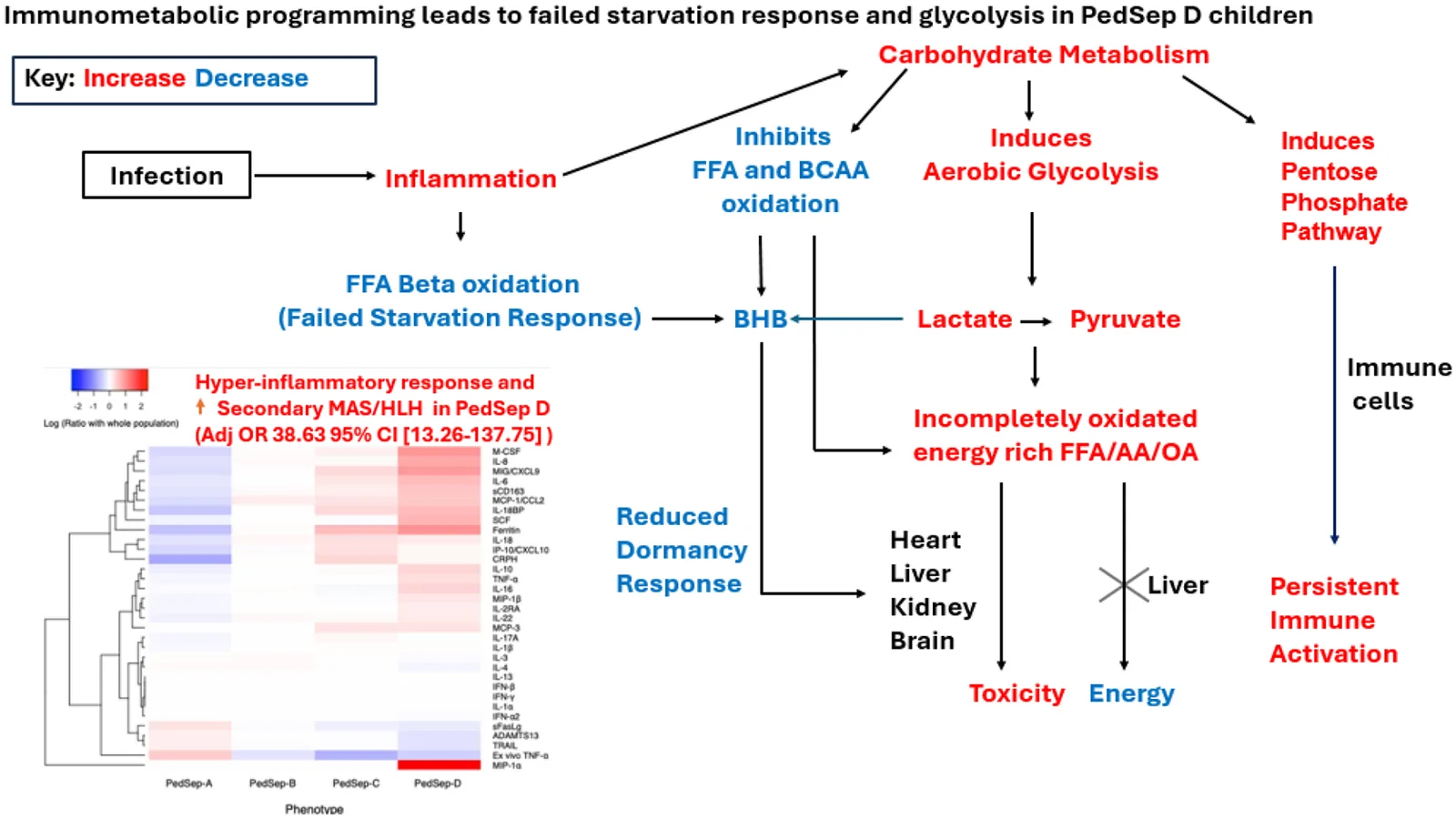
Representation of findings in this metabolomic analysis comparing vulnerable group D to tolerant group A. Red is increased and blue is decreased. A heatmap shows the log ratio of the median of 32 cytokines according to group A, B, C, and D membership assayed at the same timepoint in the same blood sample as the metabolites (12).

## Data Availability

All data generated or analyzed during this study are included in this published article [and its supplementary information files]. The datasets used and/ or analyzed during the current study are available from the corresponding author on reasonable request. The PHENOMS database is also uploaded on the NICHD sponsored DASH website.

## Abbreviations -

MOF: Multiple organ failure
PICU: Pediatric Intensive Care Unit
MAS: Macrophage activation syndrome
NIGMS: National Institute of General Medical Sciences
NICHD: National Institute of Child Health and Human Development
PHENOMS: PHENOtyping pediatric sepsis induced MOF Study
PRISM: Pediatric RISk of Mortality
SIRS: Systemic Inflammatory Response Syndrome
