# Supplementary material for: Global carbohydrate metabolism is increased and beta hydroxybutyrate decreased in sepsis vulnerable children compared to sepsis tolerant children; a cohort study
Source: Res Sq. 2026 Sep 1:rs.3.rs-10732509. Preprint. [Version 1] doi: 10.21203/rs.3.rs-10732509/v1 (PMC13684744; doi:10.21203/rs.3.rs-10732509/v1)
Supplement: Supplementary file 1 [file NIHPPRS10732509V1-supplement-1.pdf]

Supplementary Materials

Supplementary Figure 1 – Life program evolutionary theory

Supplementary Figure 2 –Hypothesis

Supplementary Figure 3 – Carbohydrate metabolism illustration ABC group and D group

Supplementary Figure 4 – Dicarboxylic acids fold differences B vs A, C vs A, and D vs A groups.

Supplementary Figure 5 - Fat metabolism illustration groups A and B

Supplementary Figure 6 – Fat metabolism illustration groups A and C

Supplementary Figure 7 – Fat metabolism illustration groups A and D

Supplemnatry Figure 8 – Failed starvation response illustration in groups C and D

Supplementary Figure 9 – Branch chain amino acid metabolites fold differences B vs A, C vs A, and D vs A groups,

Supplementary Figure 10 – Alanine, glutamate and urea cycle metabolites fold differences B vs A, C vs A, and D vs A groups.

Supplementary Figure 11 – Branch chain amino acid, glutamate, and urea cycle metabolism illustration group B vs group A

Supplementary Figure 12 – Branch chain amino acid, glutamate, and urea cycle metabolism illustration group C vs group A

Supplementary Figure 13 – Branch chain amino acid, glutamate, and urea cycle metabolism illustration group D vs group A

Supplementary Figure 14 – Full causal inference analysis

Supplementary Methods – Expanded causal inference analysis method

Life History Theory

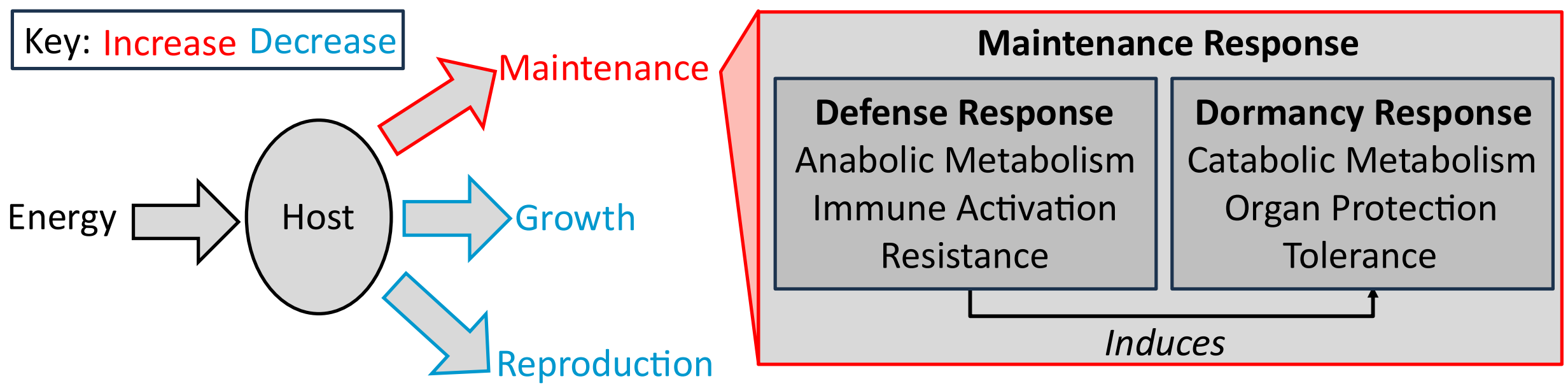

During infection the Medzhitov laboratory has proposed that maintenance programs are induced while growth and reproduction programs are suppressed. An effective maintenance program requires a balance of defense and dormancy responses.

Wang A, Medzhitov R et al. An evolutionary perspective on immunometabolism. *Science* 2019 Jan 11

Supplementary Figure 1 – Life program evolutionary theory

Hypothesis

We tested the hypothesis that children with sepsis who are most vulnerable to adverse outcome with sepsis have a dysregulated maintenance response with increased defense and decreased dormancy Immunometabolism responses.

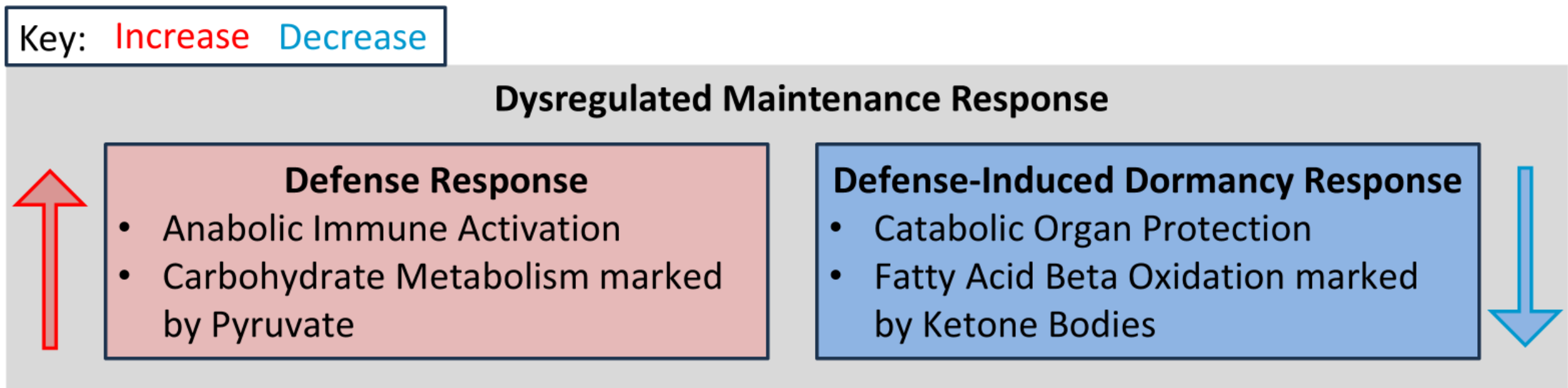

Wang A., Medzhitov R, et al Opposing effects of fasting metabolism on tissue tolerance in bacterial and viral inflammation. *Cell* 2016 Sep 8

Supplementary Figure 2 – Dysregulated maintenance program hypothesis in vulnerable sepsis

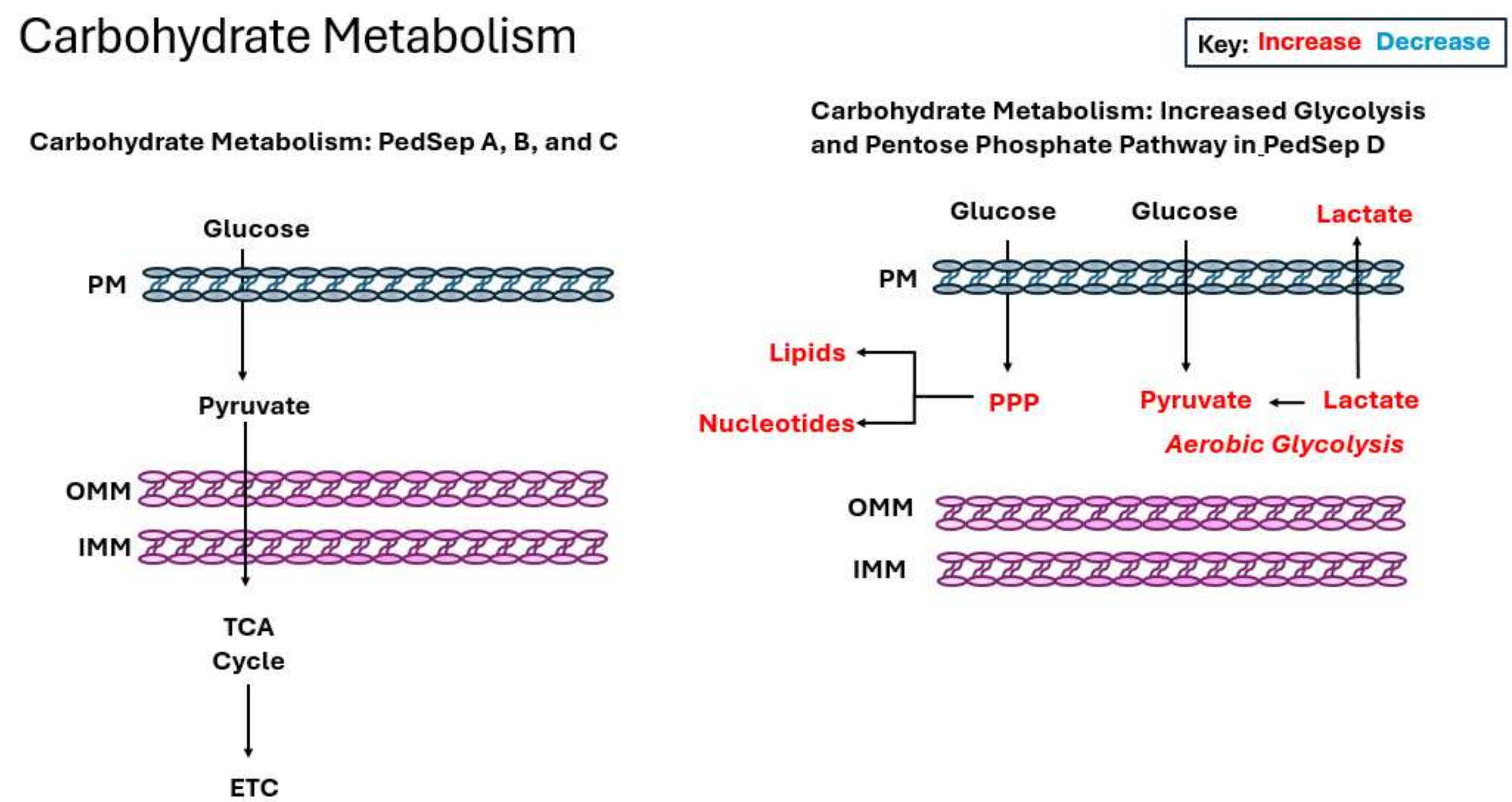

Supplementary Figure 3 – Carbohydrate metabolism illustration PedSep A, B, C and D (See Figure 2). PM – Plasma membrane; OMM - Outside mitochondrial membrane; Inside mitochondrial membrane

Pathway	Sub Pathway	Dicarboxylic Acids	B vs A	C vs A	D vs A
Lipid	Fatty Acids	glutarate (C5-DC)	1.54	0.90	2.90
		4-hydroxy-2-oxoglutaric acid	0.87	0.81	1.81
		adipate (C6-DC)	1.15	0.60	2.30
		2-hydroxyadipate	2.17	1.46	8.87
		3-hydroxyadipate	2.50	0.78	3.76
		3-methyladipate	1.50	1.81	15.41
		maleate	1.28	1.21	2.61
		pimelate (C7-DC)	2.08	1.30	5.36
		heptenedioate (C7:1-DC)*	1.15	1.65	4.43
		suberate (C8-DC)	1.31	0.69	2.90
		azelate (C9-DC)	0.95	0.93	1.12
		sebacate (C10-DC)	0.63	0.19	1.02
		decadienedioic acid (C10:2-DC)**	1.91	1.70	5.96
		dodecanedioate (C12-DC)	0.93	0.29	3.77
		3-hydroxydodecanedioate*	2.56	0.67	9.28
		dodecenedioate (C12:1-DC)*	1.97	0.77	4.84
		dodecadienoate (12:2)*	1.04	0.92	1.15
		tridecenedioate (C13:1-DC)*	1.35	1.90	6.22
		tetradecanedioate (C14-DC)	1.96	1.09	7.28
		tetradecadienedioate (C14:2-DC)*	1.94	1.44	3.83
		hexadecanedioate (C16-DC)	2.61	1.50	5.45
		hexadecenedioate (C16:1-DC)*	2.67	1.50	5.39
		octadecanedioate (C18-DC)	1.83	1.35	2.41
		octadecenedioate (C18:1-DC)	2.05	1.31	2.67
		octadecadienedioate (C18:2-DC)*	2.00	1.41	3.35
		eicosanedioate (C20-DC)	0.87	0.93	1.51
		eicosenedioate (C20:1-DC)*	0.93	1.42	1.78
		docosadioate (C22-DC)	0.93	0.87	1.49
		3-carboxy-4-methyl-5-propyl-2-furanpropanoate (CMPF)	1.73	2.81	4.27
		hydroxy-CMPF*	1.58	1.72	1.60
		3-carboxy-4-methyl-5-pentyl-2-furanpropionate (3-CMPFP)**	1.49	1.36	2.94
		2-hydroxysebacate	1.29	0.82	2.83

KEY
Fold Change Compared with PedSep A
<.5 .75 1 1.5 2 2.5 3<

Supplementary Figure 4 – Dicarboxylic acids fold differences in PedSep B vs A, C vs A, and D vs A groups. Red is increase and blue is decrease (Welch's t-test $p < 0.05$).

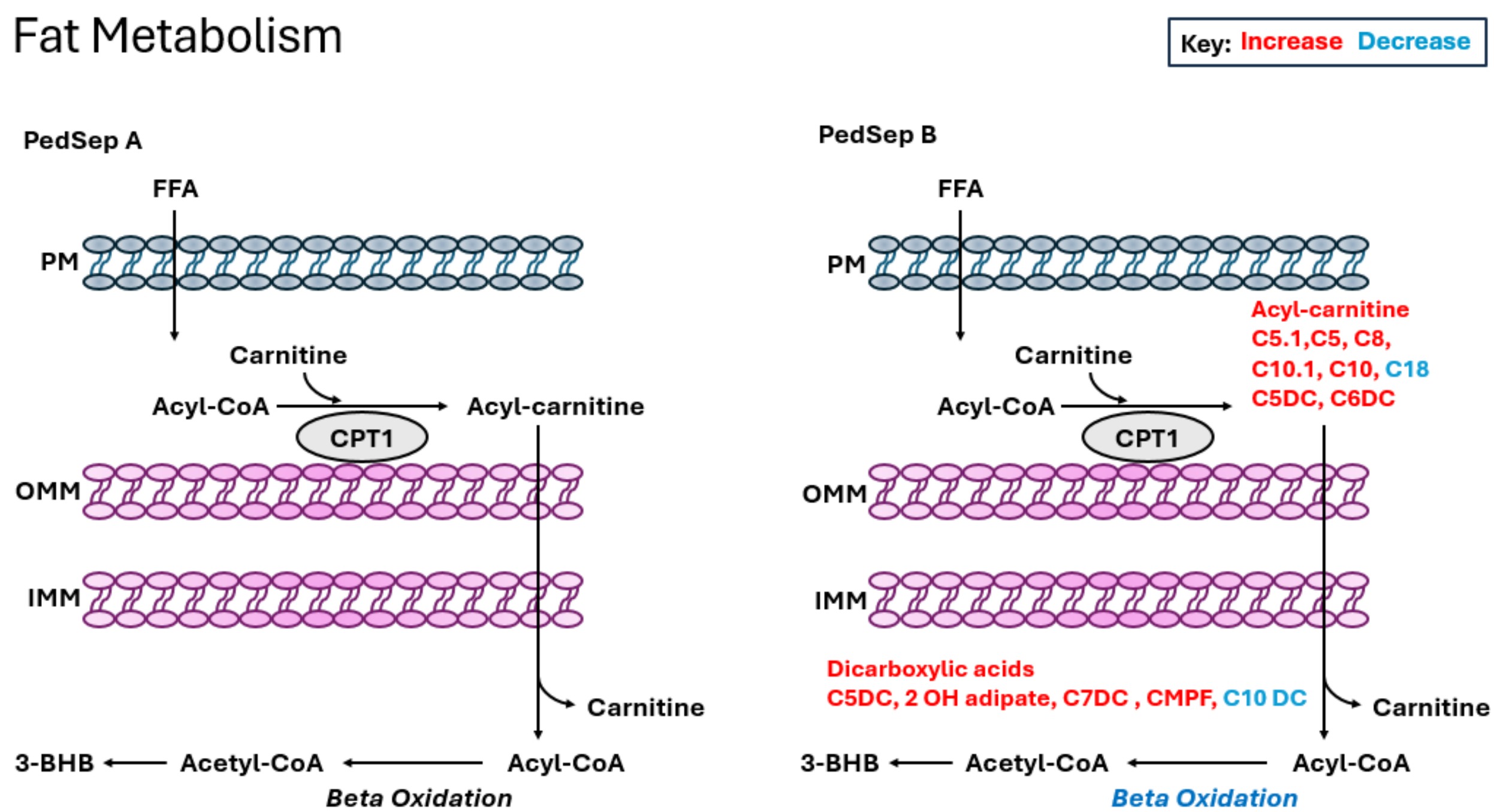

Supplementary Figure 5 - Fat metabolism illustration of PedSep A and B

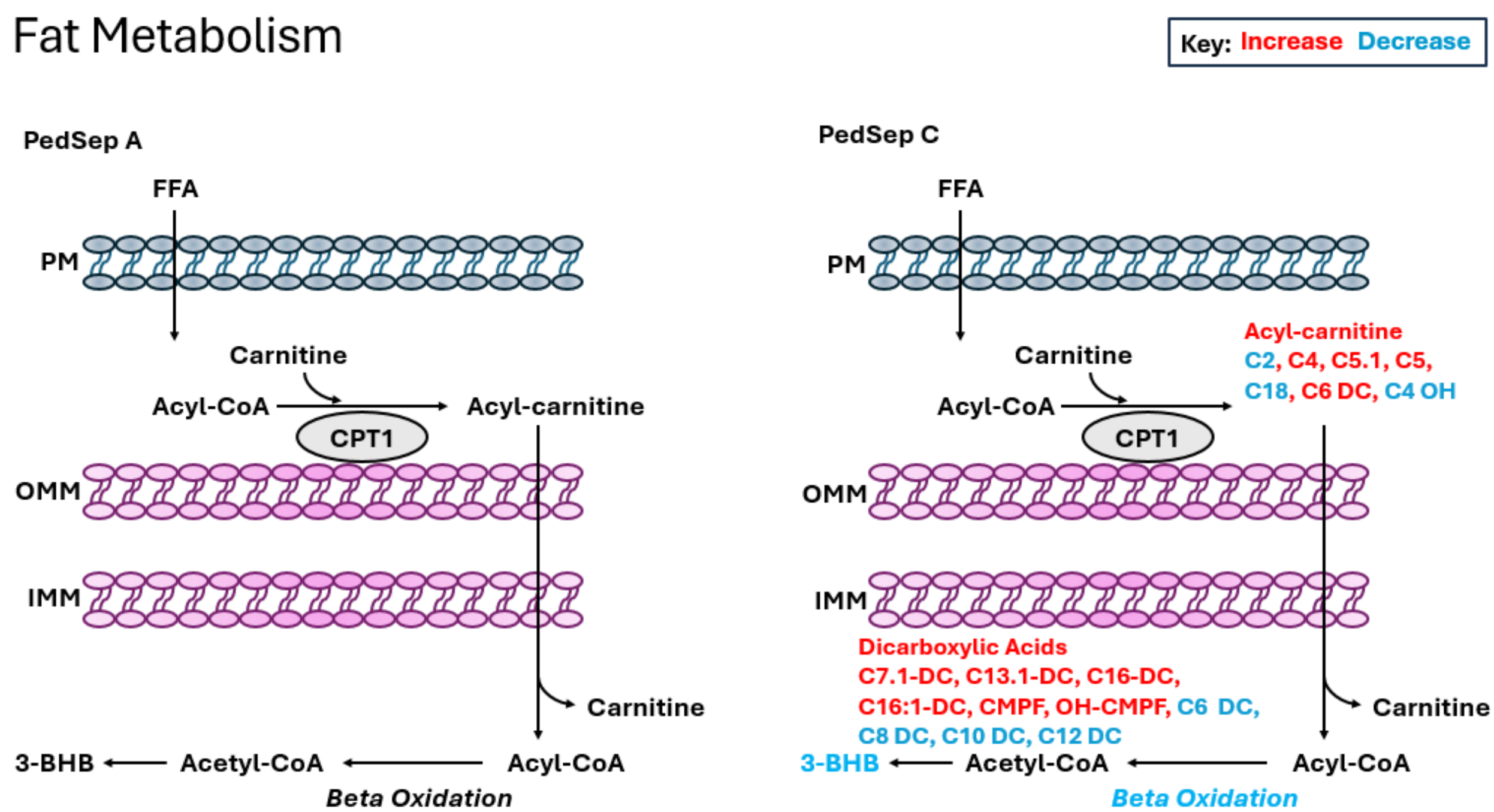

Supplementary Figure 6 – Fat metabolism illustration of PedSep A and C

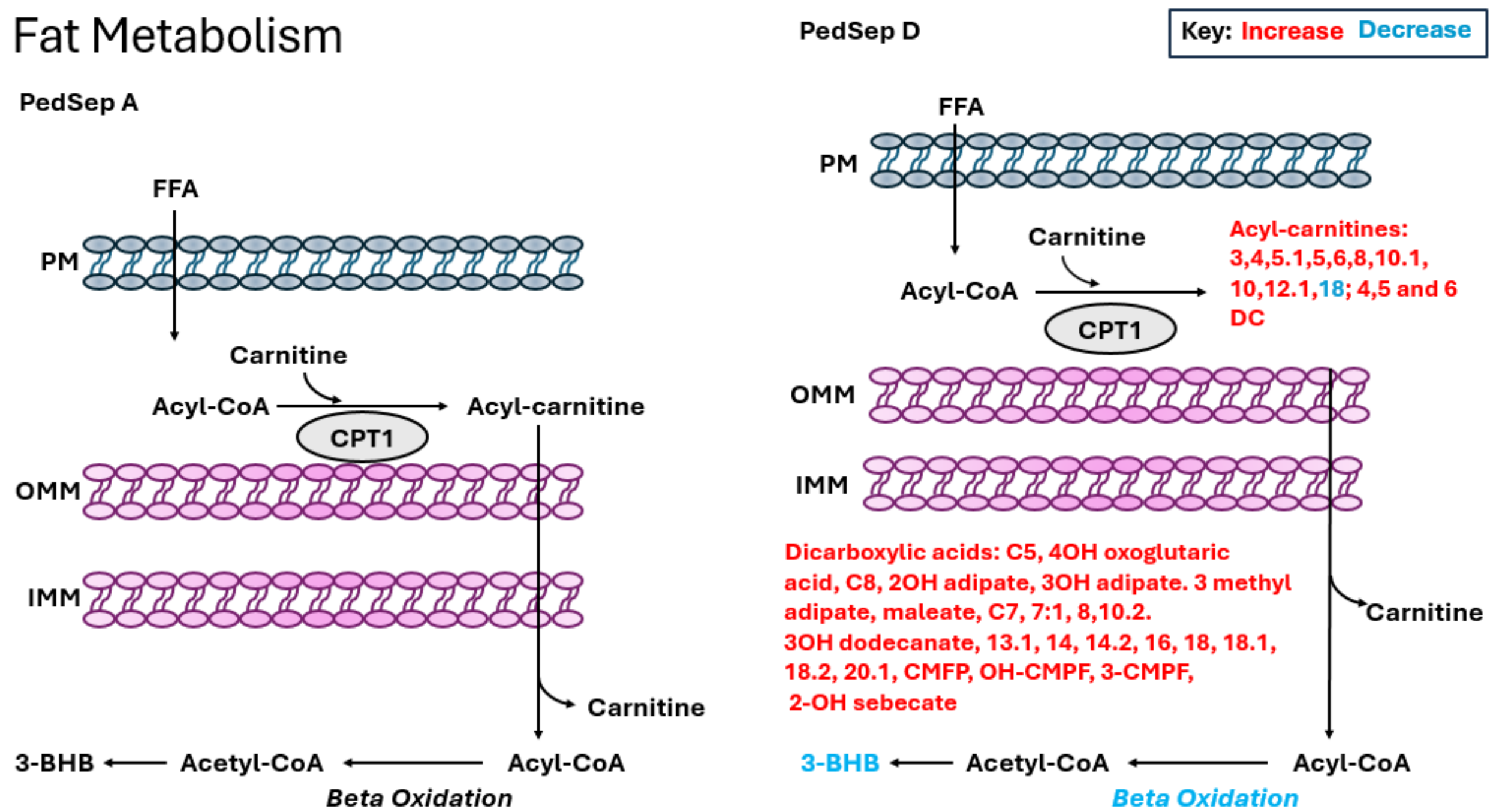

Supplementary Figure 7 – Fat metabolism illustration of PedSep A and D

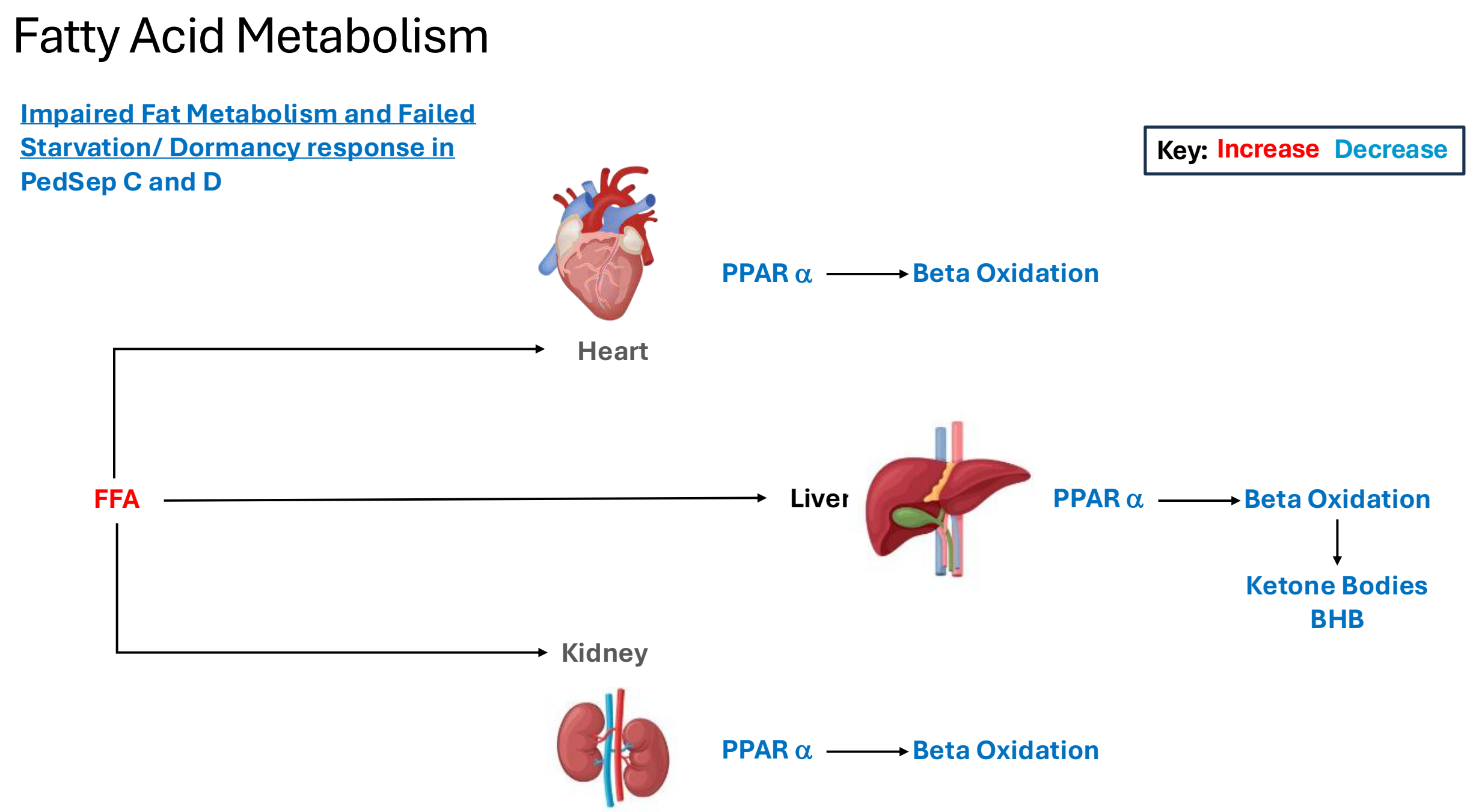

Supplementary Figure 8 – Failed starvation response illustration in PedSep C and D

KEY

Fold Change Compared with PedSep A

<.5 .75 1 1.5 2 2.5 3<

Pathway	Sub Pathway	Biochemical	B vs A	C vs A	D vs A
Amino Acid	Leucine, Isoleucine, and Valine Metabolism	leucine	1.00	1.05	1.08
		N-acetylleucine	1.31	1.31	2.52
		4-methyl-2-oxopentanoate	0.91	0.97	0.97
		alpha-hydroxyisocaproate	1.27	1.44	2.33
		2-ketocaprylate	1.25	1.01	1.17
		isovalerate (i5:0)	1.12	1.10	1.45
		isovalerylglycine	1.36	1.13	6.18
		isovalerylcarnitine (C5)	1.73	1.30	3.01
		3-methylcrotonylglycine	2.55	2.89	20.82
		beta-hydroxyisovalerate	1.37	1.23	2.60
		beta-hydroxyisovaleroylcarnitine	1.39	1.24	3.90
		3-methylglutaconate	1.98	1.69	8.46
		3-methylglutarylcarnitine (2)	2.37	2.53	11.10
		isoleucine	0.97	1.05	0.96
		N-acetylisoleucine	1.34	1.28	2.65
		3-methyl-2-oxovalerate	0.85	0.89	0.77
		2-hydroxy-3-methylvalerate	1.25	1.31	1.76
		2-methylbutyrylcarnitine (C5)	1.82	1.69	3.19
		tiglylcarnitine (C5:1-DC)	2.15	1.66	6.04
		tigloylglycine	2.40	1.75	8.01
		3-hydroxy-2-ethylpropionate	1.24	1.09	1.92
		ethylmalonate	1.30	1.00	2.24
		methylsuccinate	1.19	1.01	2.90
		methylsuccinoylcarnitine	2.41	1.37	11.44
		valine	1.03	1.12	1.30
		N-acetylvaline	1.23	1.10	2.38
		3-methyl-2-oxobutyrate	1.02	1.02	1.11
		alpha-hydroxyisovalerate	1.52	1.47	1.92
		isobutyrylcarnitine (C4)	1.45	1.56	3.57
		isobutyrylglycine	1.63	1.48	4.66
		3-hydroxyisobutyrate	1.29	1.23	2.11
		2,3-dihydroxy-2-methylbutyrate	1.85	0.86	4.77
Lipid	Fatty Acid Metabolism	butyrylcarnitine (C4)	1.26	0.80	1.37
		propionylcarnitine (C3)	0.89	0.67	5.48
		propionylglycine	0.44	0.46	1.06
		methylmalonate (MMA)	1.05	1.02	2.31

Supplementary Figure 9 – Branch chain amino acid metabolites fold differences in PedSep B vs A, C vs A, and D vs A groups. Red is increased (Welch's t-test $p < 0.05$). Blue is decreased (Welch's t-tests $p < 0.05$)

KEY
Fold Change Compared with PedSep A
<.5 .75 1 1.5 2 2.5 3<

Pathway	Sub Pathway	Biochemical	B vs A	C vs A	D vs A
		arginine	0.93	0.94	0.95
		urea	1.93	1.60	5.00
		ornithine	1.00	1.09	1.26
		3-amino-2-piperidone	1.45	1.33	3.71
		2-oxoarginine*	1.62	1.39	3.00
		citrulline	0.76	0.70	1.05
		homoarginine	1.13	1.15	0.85
		homocitrulline	0.74	0.62	1.69
		proline	0.90	0.91	1.34
	Urea Cycle; Arginine and Proline Metabolism	dimethylarginine (SDMA + ADMA)	0.99	0.94	1.57
		N-acetylarginine	1.22	1.20	2.83
		N-acetylcitrulline	1.69	1.33	11.96
		N-acetylproline	1.22	1.01	2.72
		N-delta-acetylornithine	0.95	0.89	0.99
		trans-4-hydroxyproline	0.73	0.74	1.20
		pro-hydroxy-pro	0.68	0.64	1.23
		N-methylproline	1.23	2.57	1.82
		N,N,N-trimethyl-alanylproline betaine (TMAP)	1.28	1.11	3.73
		argininate*	1.79	1.79	4.42
Amino Acid		N-acetylhomocitrulline	1.18	0.92	4.21
		glutamate	0.83	1.04	0.64
		glutamine	0.75	0.74	0.93
		alpha-ketoglutaramate*	1.35	1.23	3.72
		N-acetylglutamate	1.31	1.01	2.76
		N-acetylglutamine	1.01	0.93	2.81
	Glutamate Metabolism	4-hydroxyglutamate	0.50	0.40	0.80
		N-acetyl-aspartyl-glutamate (NAAG)	1.02	1.05	1.95
		beta-citrylglutamate	0.92	1.01	3.99
		2-pyrrolidinone	1.27	1.18	3.40
		S-1-pyrroline-5-carboxylate	1.15	1.19	2.01
		2-amino-4-cyanobutanoate	1.63	1.48	2.28
		alanine	0.97	0.96	1.24
		N-acetylalanine	1.22	1.07	2.14
		N,N-dimethylalanine	1.93	1.71	2.20
	Alanine and Aspartate Metabolism	aspartate	0.95	1.02	0.78
		N-acetylaspartate (NAA)	1.55	0.93	2.39
		asparagine	0.82	0.77	0.93
		N-acetylasparagine	1.03	0.96	4.79
		hydroxyasparagine**	1.26	1.15	2.94

Supplementary Figure 10 – Alanine, glutamate and urea cycle metabolites fold differences in PedSep B vs A, C vs A, and D vs A. Red is increased (Welch's t-test $p < 0.05$). Blue is decreased (Welch's t-tests $p < 0.05$).

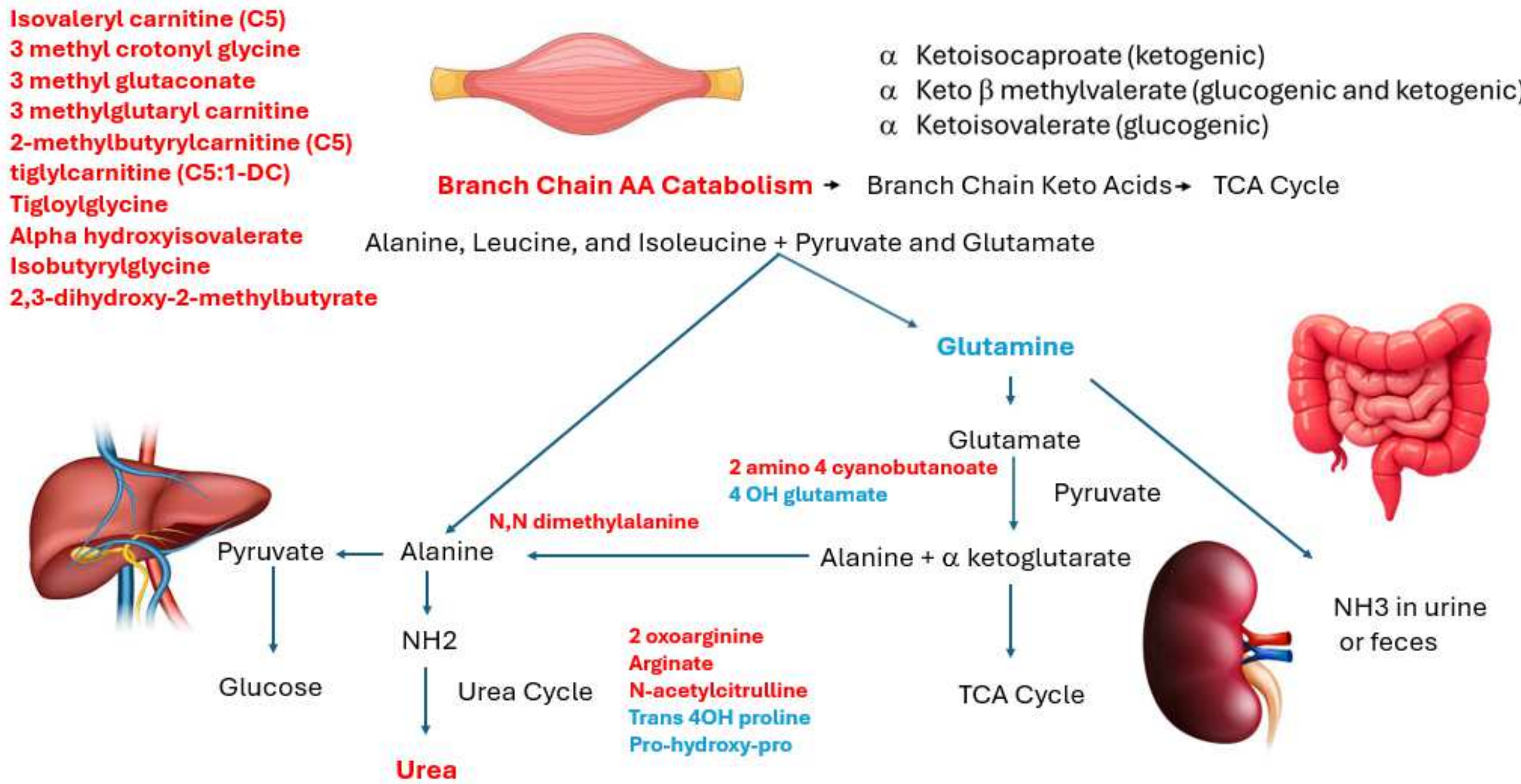

Supplementary Figure 11 – Branch chain amino acid, glutamate, and urea cycle metabolism illustration in PedSep B vs A. Red is increased. Blue is decreased.

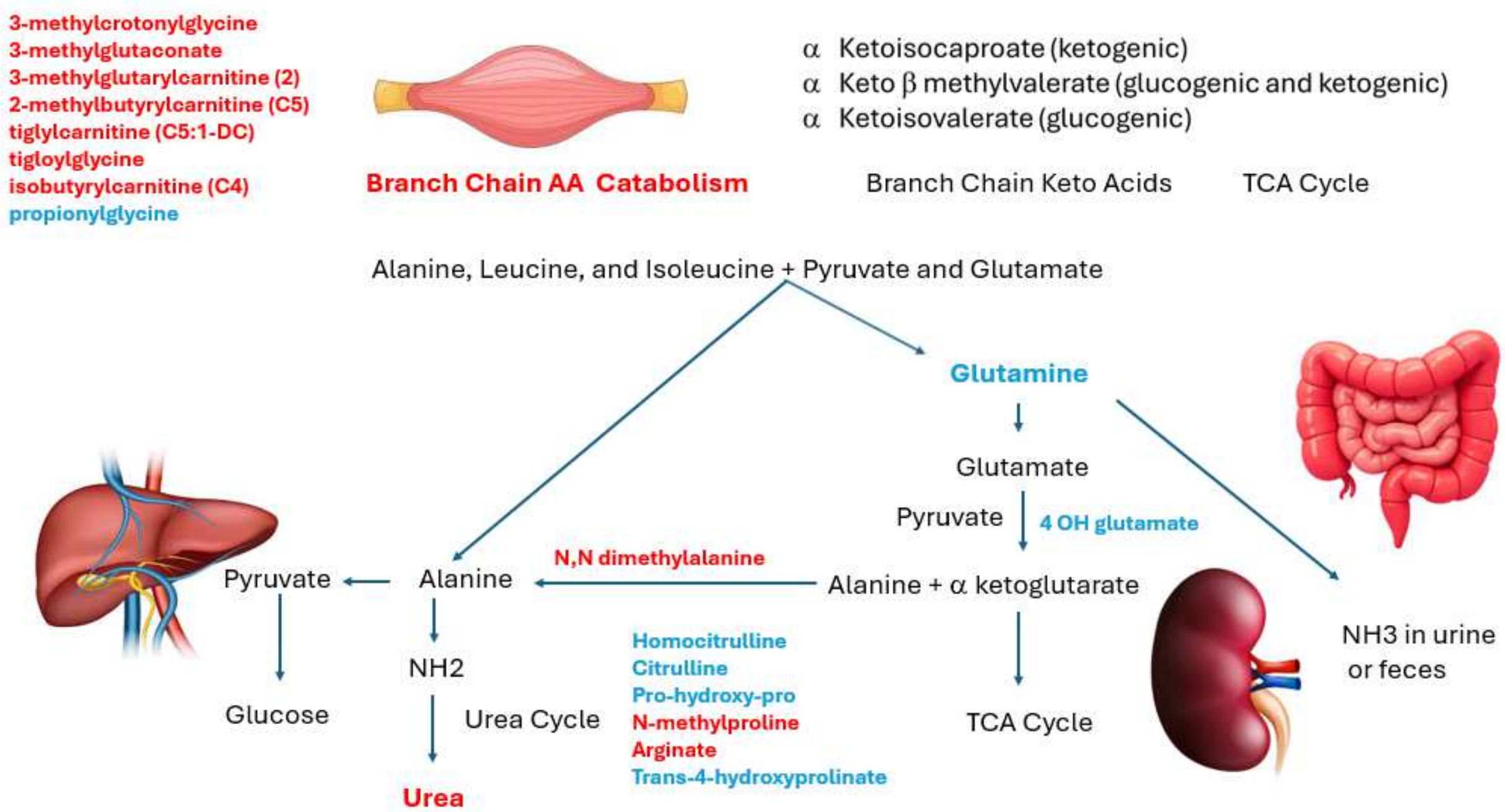

Supplementary Figure 12 – Branch chain amino acid, glutamate, and urea cycle metabolism illustration in PedSep C vs A . Red is increased. Blue is decreased.

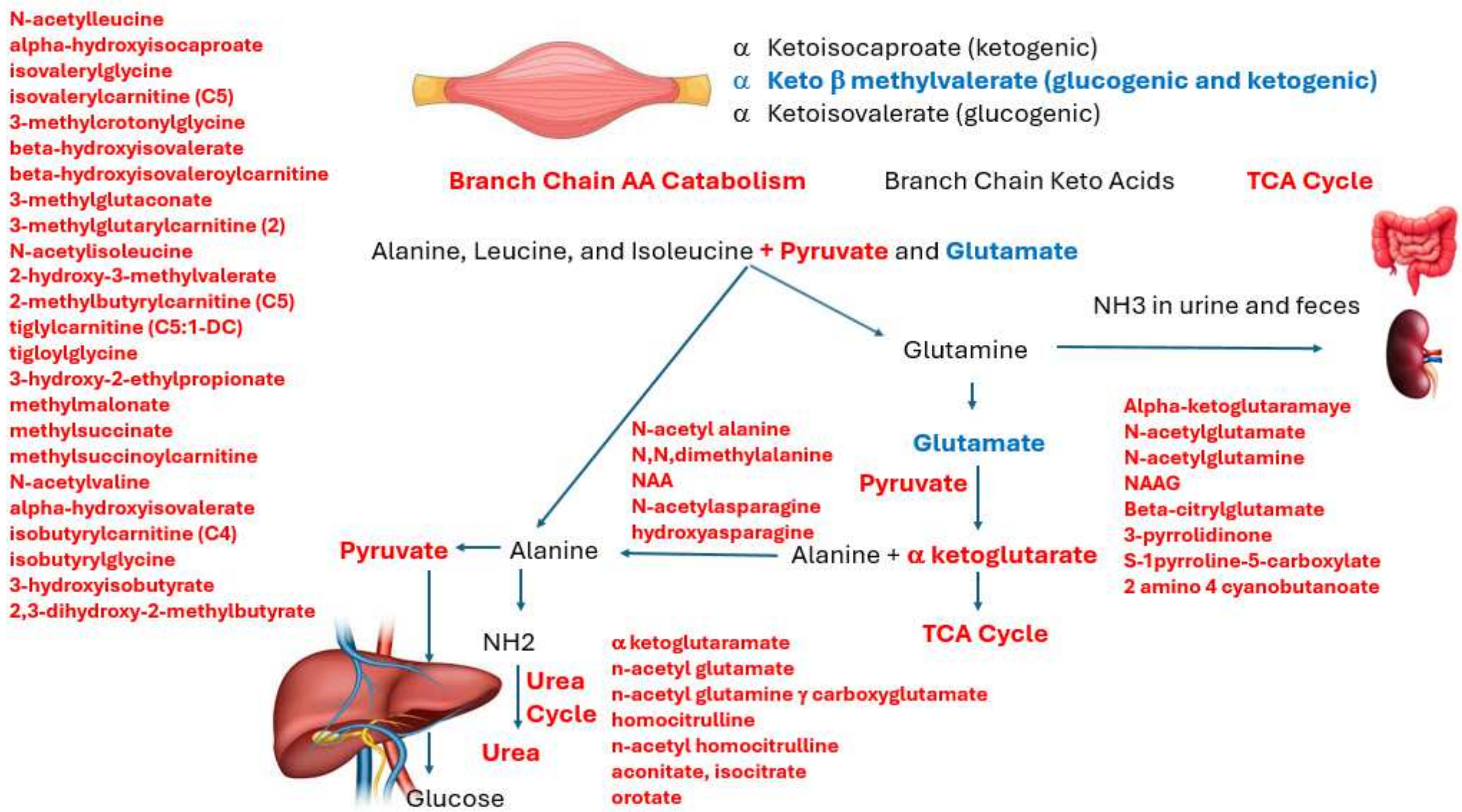

Supplementary Figure 13 – Branch chain amino acid, glutamate, and urea cycle metabolism illustration in PedSep D vs A. Red is increased. Blue is decreased.

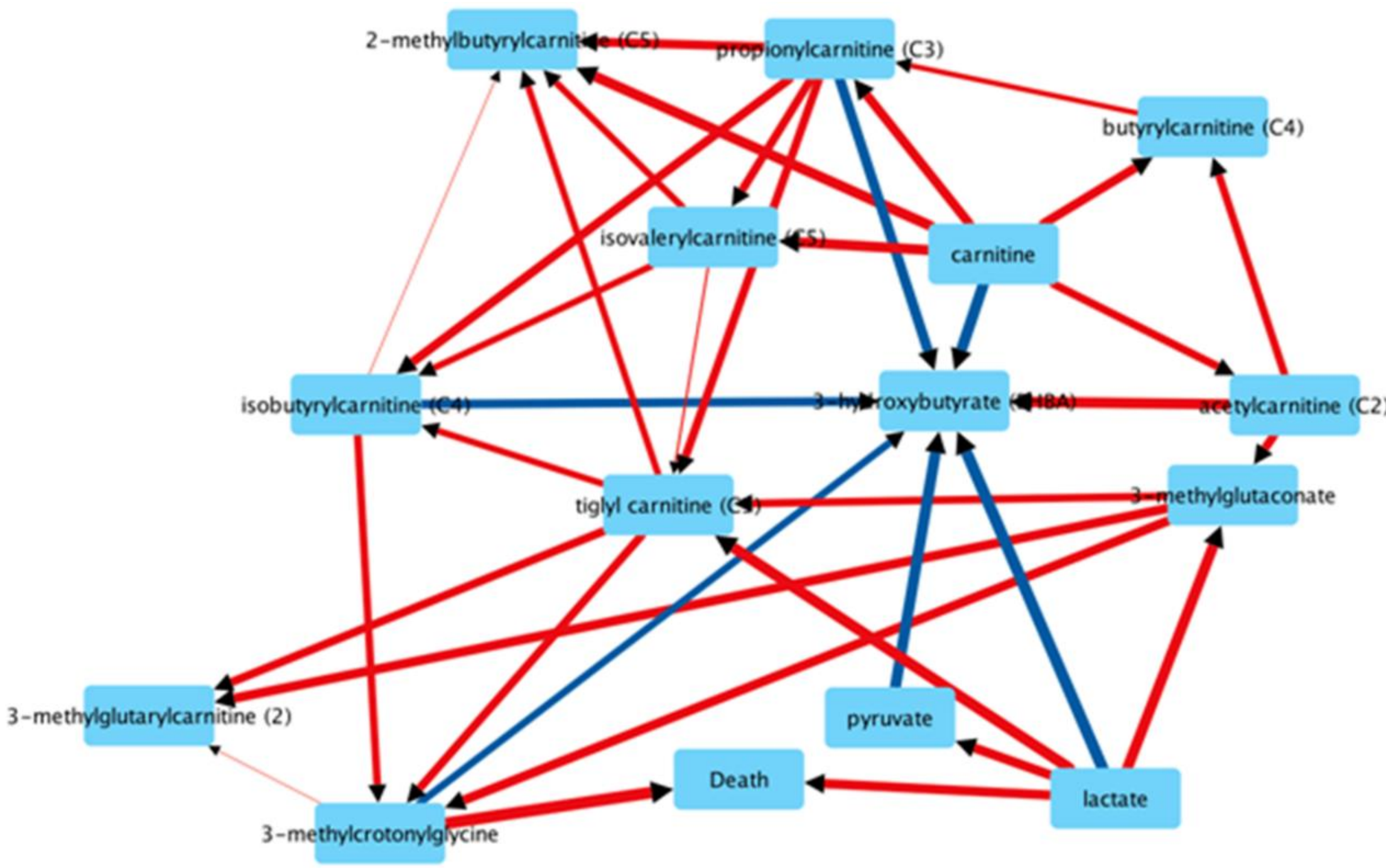

Supplementary Figure 14 - Full Causal Inference Analysis. Red arrows increase. Blue arrows decrease.

Expanded Causal Inference Analysis Methods

Identifying Potential Causal Associations Using CausalMGM and DAG

We identified potential causal associations between the metabolites and death using a mixed graphical model (MGM) algorithm called causalMGM to initiate the inference of causal associations (1). CausalMGM is an extension of Lee and Hastie's MGM that estimates a Markov random field over a set of random variables. The Markov random field for the graphical node representing a random variable is a set of all the other random variables the random variable is conditionally dependent on. Due to the conditional dependence, the Markov random field of variable T contains the minimal set of variables that are necessary to estimate T, including only the direct causes (parents), direct effects (children), and direct causes of the direct effects (spouses) of T. With the Markov random field as a focused set of variables to examine for causal associations, a graphical model of the Markov random field enables users to examine their data to identify the direct effects on a chosen variable of interest or to discover new associations between variable pairs.

Loop-Erased Random Walks as a Pathway Analysis Framework

To determine the causal directions in the graphical model (undirected graph) from a potential metabolite to death, we used random walks. Broadly, within the context of network biology, a random walk is a process where a sequence of steps is taken randomly from one node to another, going through the network to explore its structure and any potential association between features in the network (2). Recently, random walks have been used to determine causal directions in biological networks (3,4). For example, Tu et al (5) used a heuristic random walk in an integrated network to find regulatory modules in gene expression data, identifying the most likely paths

from quantitative trait loci to a candidate gene. Often in network graphs, random walks inherently have self-looping, which refers to walks that begin and end at the same node. Additionally, to explore as much of the network space as possible without informational redundancies due to self-looping, we implemented loop erasure.

To identify potentially causal pathways from metabolites to death, we implemented a loop-erased random walk (LERW) algorithm on our set of vertices and edges identified by causalMGM. In a LERW construct, any time a loop is formed the loop is immediately erased (6). To initialize paths for our random walks, we start the random walk at vertex s, setting the initial path P = [s] and label our sink node specifying where walks should end. In our setup, s = metabolite and our sink node is death. We use 500 random walks for all analyses. The network graph is weighted according to causal association effect sizes identified by the causalMGM algorithm. Larger effect size values indicate stronger causal associations.

Subnetwork Identification and Network Graph Drawings

The LERW analysis generated a list of all paths from metabolites to death. To highlight the most prominent nodes, we normalized all nodes in each pathway by the total length and aggregated them. Then, we used the mean distance to death across all the random walks as a metric to highlight which nodes were causally the closest to death. Nodes that did not get visited at least 25 times (<5%) were not included as part of the resulting subnetworks. We used Cytoscape, version 3.9.1 (Cytoscape Consortium) for the visualization of our feature phenotype causal association results (7). Arrow widths in the network graphs are proportional to the derived effect sizes from the causalMGM algorithm.

1) Sedgewick AJ, Buschur K, Shi I, et al. Mixed graphical models for integrative causal analysis with application to chronic lung disease diagnosis and prognosis. Bioinformatics. 2019; 35: 1204-12.

2) Valdeolivas A, Tichit L, Navarro C, et al. Random walk with restart on multiplex and heterogeneous biological networks. Bioinformatics. 2019; 35: 497-505.

3) Gentili M, Martini L, Sponziello, et al. Biological Random Walks: multi-omics integration for disease gene prioritization. Bioinformatics. 2022; 38: 4145-52.

4) Komurov K, White MA, Ram PT. Use of data-biased random walks on graphs for the retrieval of context-specific networks from genomic data. PLoS Comput Biol. 2010; 6: e1000889.

5) Tu Z, Wang L, Arbeitman MN, et al.. An integrative approach for causal gene identification and gene regulatory pathway inference. Bioinformatics. 2006; 22: e489-e96.

6) Wiese KJ, Fedorenko AA. Field theories for loop-erased random walks. Nucl Phys B. 2019; 946: 114696.

7) Shannon P, Markiel A, Ozier O, et al. Cytoscape: a software environment for integrated models of biomolecular interaction networks. Genome Res. 2003; 13: 2498-2504.
